# Stock Market Reactions to COVID-19 Pandemic Outbreak: Quantitative Evidence from ARDL Bounds Tests and Granger Causality Analysis

**DOI:** 10.3390/ijerph17186729

**Published:** 2020-09-15

**Authors:** Ștefan Cristian Gherghina, Daniel Ștefan Armeanu, Camelia Cătălina Joldeș

**Affiliations:** Department of Finance, Bucharest University of Economic Studies, 6 Piata Romana, 010374 Bucharest, Romania; darmeanu@yahoo.com (D.Ș.A.); joldes.catalina@yahoo.com (C.C.J.)

**Keywords:** COVID-19, stock market, ARDL model, Granger causality

## Abstract

This paper examines the linkages in financial markets during coronavirus disease 2019 (COVID-19) pandemic outbreak. For this purpose, daily stock market returns were used over the period of December 31, 2019–April 20, 2020 for the following economies: USA, Spain, Italy, France, Germany, UK, China, and Romania. The study applied the autoregressive distributed lag (ARDL) model to explore whether the Romanian stock market is impacted by the crisis generated by novel coronavirus. Granger causality was employed to investigate the causalities among COVID-19 and stock market returns, as well as between pandemic measures and several commodities. The outcomes of the ARDL approach failed to find evidence towards the impact of Chinese COVID-19 records on the Romanian financial market, neither in the short-term, nor in the long-term. On the other hand, our quantitative approach reveals a negative effect of the new deaths’ cases from Italy on the 10-year Romanian bond yield both in the short-run and long-run. The econometric research provide evidence that Romanian 10-year government bond is more sensitive to the news related to COVID-19 than the index of the Bucharest Stock Exchange. Granger causality analysis reveals causal associations between selected stock market returns and Philadelphia Gold/Silver Index.

## 1. Introduction

With globalization, urban sprawl, and ecological transformations, contagious disease outbursts turned out to be worldwide risks demanding a joint reply [1]. According to the International Monetary Fund (IMF), coronavirus disease 2019 (COVID-19) generated an economic crisis different from the others [2] for the reason that it is much more multifaceted (interconnections between the economy and the health system), uncertain (the related treatment is established gradually, alongside the measures concerning how to streamline isolation and the means to start over the economy), and has a worldwide character. Both supply and demand reductions occur since individuals work and consume lower, whereas companies diminish their productivity and investment [3]. Hence, Erokhin and Gao [4] explored 45 developing states and established that food security status of individuals and the strength of food supply chains are impacted by COVID-19.

Consequently, governments have taken unprecedented actions, respectively fiscal measures figuring to around $8 trillion, whereas central banks injected liquidity getting up to over $6 trillion [5]. The IMF has implemented exceptional measures by doubling its emergency loaning volume to $100 billion and deferring debt outflows for poor nations [6]. Preparing for the economic recovery raised a number of issues such as the way to maintain fiscal stimulus and unconventional monetary policy, managing high unemployment, low interest rates, and preserving financial stability [7]. Hence, Narayan, et al. [8] exhibited that stimulus packages enhanced stock returns in Canada, UK, and USA, but travel bans improved stock returns merely in Canada and Germany.

The crisis caused by novel coronavirus severely limited broad economic activity [9]. Barro, et al. [10] contended that related economic failures are equivalent to those last registered throughout the global Great Recession of 2008–2009. In a more pessimistic view, World Bank [11] forecasted that the worldwide health crisis is driving the worst global recession since World War II. Hence, Fernandes [12] estimated for 30 countries that a decline in gross domestic product of −2.8% will occur in 2020. As well, Gormsen and Koijen [13] predicted that economic growth will decrease by 3.8% in the United States and by 6.3% in the European Union. Likewise, Estrada, et al. [14] claimed that the potential growth of China would be reduced by 0.45%, respectively an undesirable impact of about three times higher than the outcome of Severe Acute Respiratory Syndrome (SARS).

COVID-19 is an emblematic black swan case, its incidence, expansion, and dissolution, as well as the complexity, range, and strength of its influence, are all indefinite [15]. Thus, the substantial insecurity of the outbreak and its related economic damages has entailed markets to become extremely unstable and changeable [16]. On March 16, 2020, Chicago Board Options Exchange Volatility Index (VIX) closed at the uppermost level since its inauguration [17]. Gold registered the highest level since January 2013 [18], but has been particularly variable since mid-February [19]. Nevertheless, the safe haven standing of gold vanished during corona crisis because its prices shifted in tandem with the stock markets of the ten largest economies [20,21]. As well, on April 20, 2020, traders tried to avoid physical possession of oil and massively sold oil futures contracts, sending them into negative region for the first time in history [22]. Salisu, et al. [23] found that a 1% drop in crude oil price returns rises the likelihood of registering undesirable stock returns before the pandemic proclamation. Consequently, there was acknowledged that the coronavirus pandemic has weakened oil demand and there is not enough storage space for overproduction of oil in the United States (for instance, nearly 85% of global onshore storage was filled) [24]. Investors liquidated the May futures contracts that matured on Tuesday (April 21, 2020), the price of West Texas Intermediate (WTI) oil registering the value of –37.63 dollars/barrel, at the end of the day [25].

Recent studies focused on the impact of coronavirus on various measures such as exchange rate [26], financial volatility [27,28], stock returns [29,30,31,32,33,34], corporate bonds [35] or Eurobonds [36], oil price [37], or economic policy uncertainty [38], alongside employing various methods towards assessing the diffusion of the virus [39,40] or assessing the source of health security [41,42]. We contribute to this growing literature by exploring the associations in stock markets throughout COVID-19 pandemic outbreak. First, we explore whether the Romanian stock market is impacted by the crisis generated by novel coronavirus. To the best of our knowledge, this is the first study addressing the impact of COVID-19 from both China and Italy on the Romanian capital market and the 10-year Romanian bond. Nations in Eastern Europe have circumvented huge virus occurrences than those registered in other parts of the continent [43] such as Italy, United Kingdom, Spain, or France. Nonetheless, Romania is one of the most affected country in the region since many of its citizens get back from Italy and Spain [44]. Many developing nations depending on overseas revenue in form of a mixture of commodity exports, tourism, and remittances are expected to fail due to liquidity scarcity and lack of tax revenues [45]. Eissa [46] highlighted disparities in health expenditures per capita, the highest levels being registered in North America and Western Europe, but the lowest in West, Central, and East Africa. Although remarkable fiscal-budgetary instruments have been implemented by many European governments (e.g., 50 per cent of GDP in Italy, 28 per cent in Germany, 19 per cent in France, 12 per cent in Poland, 11 per cent in Spain, 6.5 per cent in Serbia), Romania ensured a fiscal assistance of just 3.5 per cent of GDP [47]. Secondly, our research investigates the causalities among COVID-19 and major stock market returns, as well as between pandemic measures and several commodities.

The rest of this manuscript proceeds as follows. Section 2 reviews prior studies. Section 3 discusses the sample and quantitative methods. Section 4 focuses on empirical outcomes. Final section presents the conclusions and the main policy implications.

## 2. Related Literature

### 2.1. Prior Research Regarding the Economic and Financial Consequences of COVID-19

The COVID-19 contagion triggered a failure in worldwide stock markets resulting in an unpredictable setting with critical liquidity levels [48]. Therewith, substantial contagion between nations was noticed by Hafner [49] attributable to noteworthy serial and spatial autocorrelations. Giudice, et al. [50] noticed that current pandemic affected housing values, whereas Babuna, et al. [51] emphasized that insurance industry registered losses.

Beck, et al. [52] investigated ten emerging markets and found that most of companies were harmfully influenced by COVID-19, whereas Haroon and Rizvi [53] explored 23 emerging markets and provided support that reducing (growing) course of coronavirus cases is related with enhancing (worsening) liquidity in financial markets. In a similar vein, Baig, et al. [54] claimed that community panic, alongside constraints and quarantine drive the cash shortage and uncertainty of the markets. Erdem [55] investigated stock market indices of 75 nations and supported that markets are negatively influenced by the pandemic. Therefore, the coronavirus health calamity switched into a wider economic and financial disaster [56], marked by decline in business profitability and employment, alongside an upsurge in debt [57]. To these concerns are added the ongoing challenges like stimulating trade, fintech, digital transformation, and combating climate change.

Since the SARS-CoV-2 virus is spreadable and migrations occurs, current pandemic outbreak affect many nations worldwide, along with their stock markets [58]. Hence, Shehzad, et al. [59] documented that conditional variance of stock markets from Europe and USA is huge throughout the period of COVID-19 as related to the Global Financial Crises (GFC) of 2007–2009. Estrada, et al. [60] explored ten major stock markets worldwide and cautioned that the effects of SARS-CoV-2 crisis may engender comparable impairment of the Crisis 1929, also being estimated a period between 9 and 12 months for recovery. Mishra, et al. [61] revealed that all Indian stock market returns were negative during COVID-19 as compared with contemporary main structural changes such as demonetization and implementation of goods and services tax. In contrast, Bhuyan, et al. [62] exposed that stock market returns of the SARS diseased nations displayed substantial rise related to the pre-SARS stage. Baltussen and Vliet [63] concluded that through the recovery period in the aftermath of Spanish Flu contagion small caps showed the strongest performance. Likewise, Ding, et al. [64] revealed that stock price decrease was lesser for companies showing pre-2020 funds, with a minor contact with the virus over international supply chains and clients places, many corporate social responsibility (CSR) actions, and fewer entrenched directors. Singh [65] argued that investors are focused on environmental, social, and governance (ESG) portfolio since it centers on the long-term sustainability of corporations. In addition, Palma-Ruiz, et al. [66] documented for a sample of 35 IBEX-35 companies that investors are more oriented towards ESG features. Therefore, Pástor and Vorsatz [67] recommended funds with high sustainability ratings, suggesting the opinion that sustainability is a requirement instead of opulence.

The occurrence of SARS-CoV-2 virus influenced the economic setting and marked investor sentiment, also triggering stock price fluctuations [15]. Yilmazkuday [68] exhibited that an upsurge in daily total fatalities due to SARS-CoV-2 will lessen the international economic activity assessed through by the Baltic Exchange Dry Index. Ru, et al. [69] claimed that investors from nations with prior knowledge of comparable calamities respond more quickly to COVID-19 than the investors deprived of experience. Hassan, et al. [70] suggested that firms having experience with SARS or H1N1 own more positive prospects towards their capacity to handle the SARS-CoV-2 epidemic.

As regards investing strategies over the SARS-CoV-2 crisis, Ortmann, et al. [71] suggested that investors open more stock and index positions, but do not shift to safe-haven or perilous investments. Hence, Cheema, Faff and Szulczyk [20], Cheema, Faff and Szulczyk [21] advised that gold and silver lost momentum in favor of liquid and stable assets such as treasuries and the Swiss franc. Mensi, et al. [72] proved that gold and oil turned out to be more inefficient throughout the corona crisis related to the pre-pandemic period. Hence, investors can establish profitable approaches by exploiting market inefficiencies to acquire abnormal returns [73]. On the contrary, Yan, et al. [74] recommended the tourism industry, technology sector, leisure industry, and gold as suitable investments. Li, et al. [75] endorsed health sector in line with Chong, et al. [76] which suggested over SARS to buy medical stocks and sell tourism stocks. In terms of cryptocurrencies, Chen, et al. [77] argued that augmented concerns of the coronavirus caused negative Bitcoin returns and large trading volume, whereas Conlon and McGee [78] advised that it does not perform as a hedge.

With reference to the influence of the pandemic on the enterprise’s activities, Mazur, et al. [79] contended that companies reply in various means to the COVID-19 revenue shock because many sectors were locked throughout the quarantine stage. Hence, Xiong, Wu, Hou and Zhang [9] evidenced that companies belonging to sectors that are exposed to the pandemic have significantly lower cumulative abnormal returns, but enterprises with good financial conditions endure less opposing effect of the disease. Nguyen [80] established that energy segment experienced the utmost abnormal negative returns amid all sectors. Fallahgoul [81] established that the financial segment is the most doubtful, whereas health is the most hopeful over the COVID-19 pandemic. He, Sun, Zhang and Li [15] claimed that manufacturing, information technology, education and health-care Chinese sectors remained stable to COVID-19. Gu, et al. [82] found that Chinese manufacturing sector was hardly hit by corona crisis, but construction, information transfer, computer services and software, and health care and social work were positively influenced by COVID-19.

### 2.2. Earlier Studies towards the Impact of COVID-19 on Stock Markets

Financial markets worldwide confronted with the flight-to-safety phenomenon which engendered a severe deterioration in asset appraisals and amplified volatility around the world [11]. Baker, Bloom, Davis, Kost, Sammon and Viratyosin [30] stressed that there was no prior illness that determined such daily stock market jumps. Albulescu [83] emphasized that the fatality rate has a positive and very significant influence on financial volatility, whereas Albuquerque, Koskinen, Yang and Zhang [31] found that green stocks are highly valued and register lower volatility and larger trading volumes than the rest of stocks.

Markets are a function of government, hence responding reliant on authority reply [84]. Alfaro, Chari, Greenland and Schott [32] confirmed that a doubling of projected contaminations is linked with a 4 to 11 percent deterioration of aggregate market value. Alber [85] showed that stock market return is influenced by COVID-19 cases more than deaths, as well as by aggregate measures more than new ones. However, attributable to local features, the influence of novel coronavirus may diverge across equity markets [86]. Onali [33] revealed that variations in the amount of cases and deaths in the USA and other highly impacted nations by the coronavirus do not influence stock market returns out of USA, except the number of cases for China. The spread of COVID-19 globally driven an upsurge of yields on sovereign securities more than proportionally in developing and emerging states [36]. Nozawa and Qiu [35] noticed that corporate bonds supplied by companies showing a strong link with China respond more to the quarantine of Wuhan at early 2020. Hence, M.Al-Awadhi, Alsaifi, Al-Awadhi and Alhammadi [29] concluded that the COVID-19 disease negatively influence stock market returns of the companies covered in the Hang Seng Index and Shanghai Stock Exchange Composite Index. Adenomon, Maijamaa and John [34] strengthened that the coronavirus disease negatively influences the stock returns in Nigeria.

On the contrary, there was proved that everyday cases of new contagions have a low adverse effect on the crude oil quotations in the long-term [37]. Albulescu [38] explored whether the COVID-19 and crude oil influence the economic policy uncertainty of the United States and observed no impact when considering the global coronavirus data, but a positive effect when assessing the condition outside China. Sharif, et al. [87] established a unique responsiveness of stock market of USA, related economic policy uncertainty, and geopolitical risk to the joint shocks of the coronavirus and oil instability. For the case of Colombia, Cardona-Arenas and Serna-Gómez [26] argued that the depreciation of national currency against the dollar commenced after the diagnosis of the initial positive coronavirus case which determined a rise in global oil value.

Pavlyshenko [39] argued that varied turmoil exerts distinct influence on the similar assets. Hence, Mamaysky [88] exhibited that VIX is most Granger caused by the news even if the other asset kinds are also Granger caused by the news.

Due to reduced level of economic growth and deficiency of capital influxes, emerging markets show inadequate funds to handle the pandemic and thus are likely to undergo worst [86]. Hence, we postulate the following research hypotheses:

**Hypothesis** **1** **(H1).** *The stock market index of the Bucharest Stock Exchange is negatively affected by the number of new cases and new deaths due to COVID-19 in China and Italy.*

**Hypothesis** **2** **(H2).** *The Romanian 10-year bond yield is negatively affected by the number of new cases and new deaths due to COVID-19 in China and Italy.*

## 3. Empirical Framework

### 3.1. Sample and Variables

Daily stock market returns over the period 31 December 2019–20 April 2020 were collected for the following economies: United States (USA), Spain (ES), Italy (IT), France (FR), Germany (DE), United Kingdom (UK), China (CH), and Romania (RO). The selected measures are depicted in Table 1. Alike Lyócsa, et al. [66], the timespan was selected since over the beginning of the corona disaster, the value of the market dropped, whereas insecurity in the market amplified severely.

In addition, we have included a wide range of variables that allow us to achieve our goal, such as COVID-19 measures, commodities, currencies, and 10-Year government bond spreads.

### 3.2. Quantitative Methods

In order to gain insights towards the linkages in stock markets during COVID-19 pandemic outbreak, we will use the autoregressive distributed lag (ARDL) model similar Albulescu [37,38], Erokhin and Gao [4], as well as Granger causality test alike Mamaysky [88]. Checking for unit root in ARDL approach is not fundamental in as much as it can examine for the occurrence of cointegration among a set of variables of order I(0) or I(1) or a mixture of them. Hence, the leading benefit of ARDL model consist in its versatility. However, the ARDL methodology impose that no variable should be integrated of second order or I(2). Therefore, in line with prior research [26,34,59], the augmented Dickey–Fuller (ADF) test will be applied for unit root testing. The null hypothesis of the ADF test claims the presence of unit root in the time series.

The ADF test involves estimating the following equation:

(1) $$∆\omega_{t}=\alpha +\beta t+{q\omega }_{t}+\sum_{j=1}^{k}\gamma_{j}∆\omega_{t-j}+\varepsilon_{t},\;t=1,\ldots ,T$$

where t denotes the time trend, T signifies the length of the sample, while k is the length of the lag in the dependent variable.

Further, ARDL model examines the long and short-term cointegration, being specified as a sole equation framed with adaptable choice of lag extents. The general form of an ARDL (p, q) model is as follows:

(2) $$W_{t}=\mu +\beta_{0}Z_{t}+\beta_{1}Z_{t-1}+\cdots +\beta_{q0}Z_{t-q}+\delta_{1}W_{t-1}+\cdots +\delta_{p}W_{t-p}+u_{t}$$

The lag orders p and q are established by means of the Akaike Information criteria and may differ over the explanatory variables covered in our quantitative framework.

The Granger causality test can be applied to analyze the causality between variables, as in Mamaysky [88]. The null hypothesis is that w does not Granger-cause z and that z does not Granger-cause w. The following bivariate regressions will be estimated:

(3) $$z_{t}=\alpha_{0}+\alpha_{1}z_{t-1}+\cdots +\alpha_{p}z_{t-p}+\beta_{1}w_{t-1}+\cdots +\beta_{p}w_{-p}+\epsilon_{t}$$

(4) $$w_{t}=\alpha_{0}+\alpha_{1}w_{t-1}+\cdots +\alpha_{p}w_{t-p}+\beta_{1}z_{t-1}+\cdots +\beta_{p}z_{-p}+u_{t}$$

## 4. Econometric Findings

### 4.1. Summary Statistics, Correlations and Stationarity Examination

The descriptive statistics of the variables are provided in Table 2. The distributions of all stock market returns, as well as most of included commodities are negatively skewed. Thus, negative returns are more prevalent than positive returns, supporting a greater likelihood for very high losses. Kurtosis shows the thickness of the tail and highlights a high level of risk for selected stock markets, especially Spain and Italy. In addition, except EUR/CNY and Natural Gas Futures Contract 1, the Jarque–Bera test provides evidence that selected series are not normally distributed.

**Figure 1:**
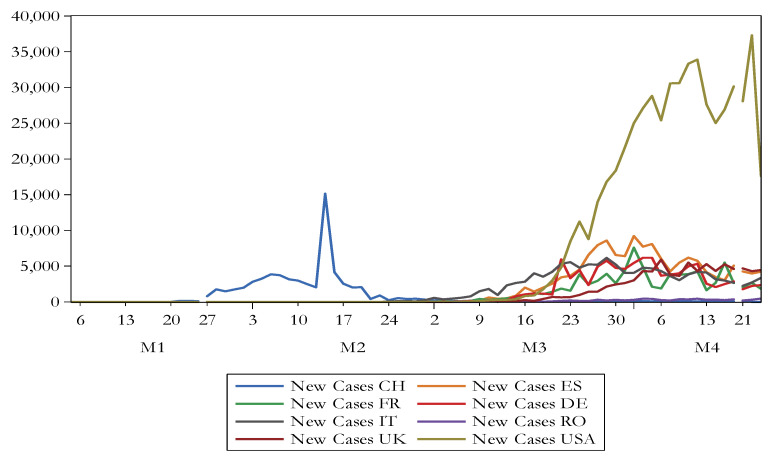
The evolution of the number of new cases due to COVID-19. Source: authors’ own work.

**Figure 2:**
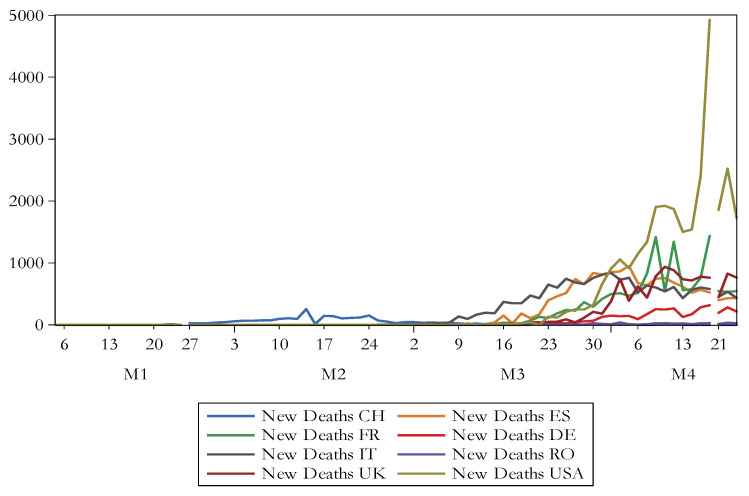
The evolution of the number of new deaths due to COVID-19. Source: authors’ own work.

Figure 1 shows the evolution of the number of new cases due to COVID-19, whereas Figure 2 reveals the progress of the number of new death due to COVID-19. There is noticed that USA registers the highest figures in this regard.

**Figure 3:**
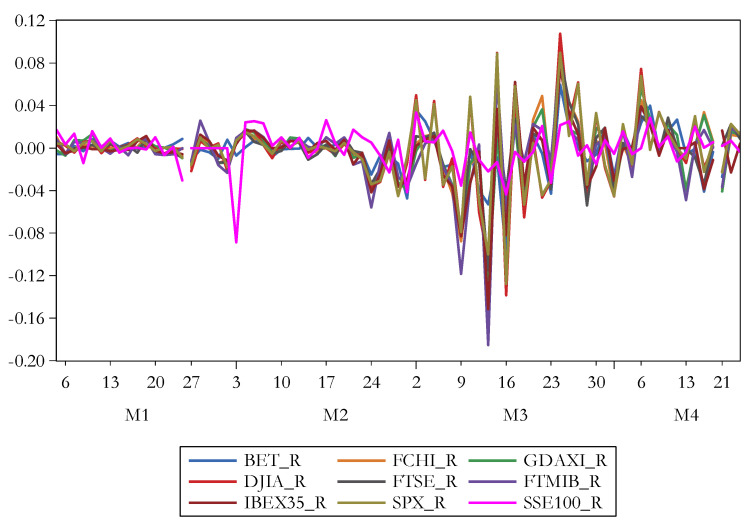
The evolution of the stock market returns. Source: authors’ own work. Notes: for the definition of variables, please see Table 1.

Figure 3 shows the evolution of stock market returns amongst the explored period. There is reinforced the significant volatility, especially for FTSE MIB on March 9, 2020 and March 12, 2020, as well as for Dow Jones Industrial Average on March 16, 2020. In the first two months of 2020, DAX declined by 10.2 percent, CAC 40 dropped by 11.2 percent, whereas FTSE 100 plunged 12.7%. In the same vein, Dow Jones throw down by 11 percent and S&P 500 by 8.6 percent. The Bucharest Stock Exchange also encountered instabilities and registered a decay of 8.6 percent [89]. Capelle–Blancard and Desroziers [90] contended that prior to February 21, stock markets disregarded the pandemic, but over February 23–March 20, the reaction to the rising number of diseased people was strong. As such, Mazur, Dang and Vega [79] emphasized that the failure of stock quotes in March 2020 marked one of the major financial market collapses in history. Baiardi, et al. [91] developed a three-regime switching model and concluded that in 2020 the most common state for the Dow Jones Industrial Average was turbulent.

**Figure 4:**
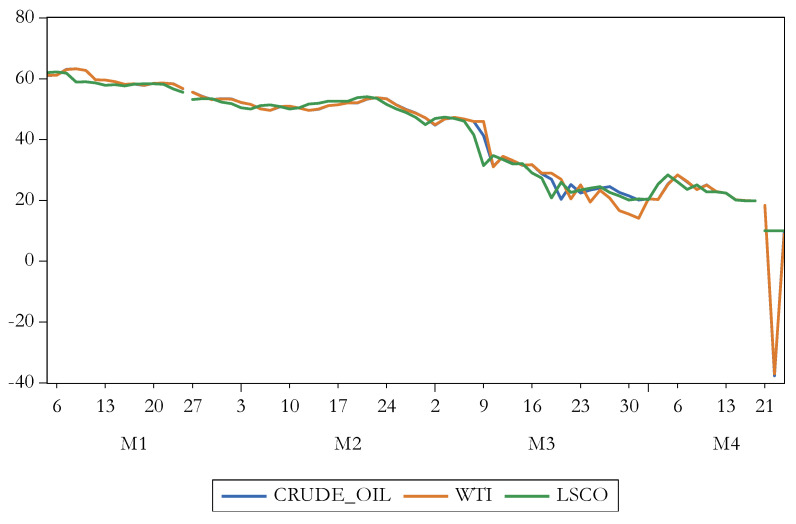
The evolution of oil futures. Source: authors’ own work. Notes: for the definition of variables, please see Table 1.

**Figure 5:**
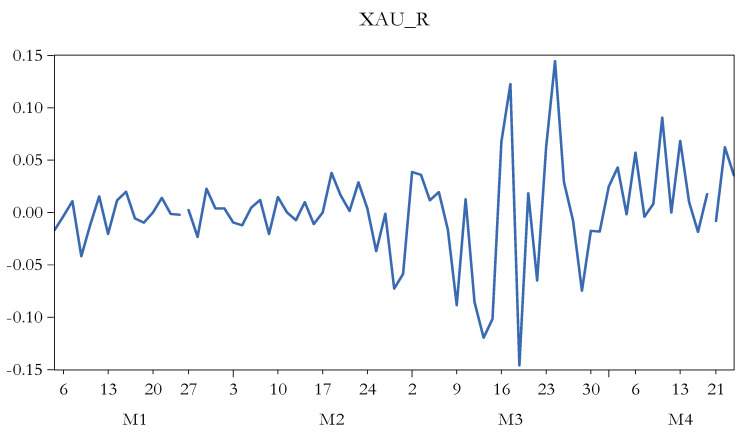
The evolution of Philadelphia Gold/Silver Index returns. Source: authors’ own work. Notes: for the definition of variables, please see Table 1.

Figure 4 reveals the evolution of oil futures. There is noticed the sharp decline registered on 21 April 2020. Figure 5 shows the progress of Philadelphia Gold/Silver Index returns. Therewith, high volatility is prevailing.

Table 3 reveals the correlations among selected variables. There are acknowledged high negative correlations (below −0.7) between the number of new cases and new deaths due to COVID-19 in Italy and crude oil, WTI, as well as NYMEX light sweet crude oil. In case of the number of new cases and new deaths due to COVID-19 in China, there are not recorded high correlations with the included measures. Therewith, high positive correlations (over 0.7) are registered amongst the stock market returns, except SSE 100 (China).

Non-stationary variables lead to inadequate results, which means insignificant results. The verification of the stationarity of the selected data is performed through ADF stationarity test. This test is most commonly used to confirm the stationarity of a data series.

Table 4 shows the results of the ADF test at the level and in the first difference, as well as the level of integration of the stock indices.

The outcomes of ADF test provide support that all covered stock indices are stationary at the first difference, showing an integration order of I(1), except the stock market index from the Shanghai Stock Exchange. We also notice that the indicators related to the evolution of COVID-19 for the most affected regions, China and Italy, show a mixed integration order (I(0)and I(1)).

### 4.2. Cointegration Analysis and Long-term Relationships

After studying the stationary of the data series and due to the mixed results, we conclude that the ARDL model is the most appropriate for exploring the linkages between variables. Further, the purpose is to assess whether new cases and new deaths due to COVID-19 in China and Italy, along with Chinese and Italian stock market returns, several commodities, and currencies are related to the Romanian stock market as measured by BET index return and Romania 10-year bond yield.

The ARDL (autoregressive distributed lag) model is used especially when the variables I(0) and I(1) are integrated. For the accurate choice of the ARDL model that would allow us to research the relationships that are established between variables, it is imperative to choose the correct number of lags. Therefore, we will analyze the Akaike information criteria (AIC) to select the optimal lags for the variables included in the ARDL model.

**Figure 6:**
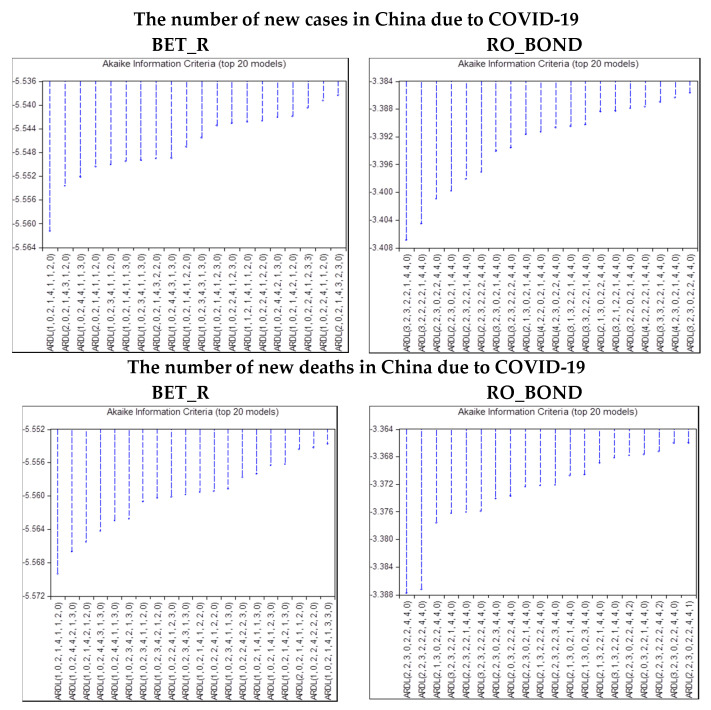
Optimal lags for the model Romania and COVID-19 (China). Source: authors’ own work. Notes: for the definition of variables, please see Table 1.

We will apply the criteria graph, which will indicate the suitable lags for the ARDL model and the lowest value is preferred. Figure 6 shows the results of criteria graph for the ARDL model that takes into account the number of new cases and new deaths in China, both for the BET stock index return and for the Romanian Government bond (10Y).

According to the results, in total, 1,562,500 ARDL model specifications were considered for each of the four cases given the information related to COVID-19 in China. The top 20 results are presented in the criteria graph.

Further, Table 5 summarizes the selected lags for the model Romania and COVID-19 (China) according to criteria graph out of Figure 6.

**Figure 7:**
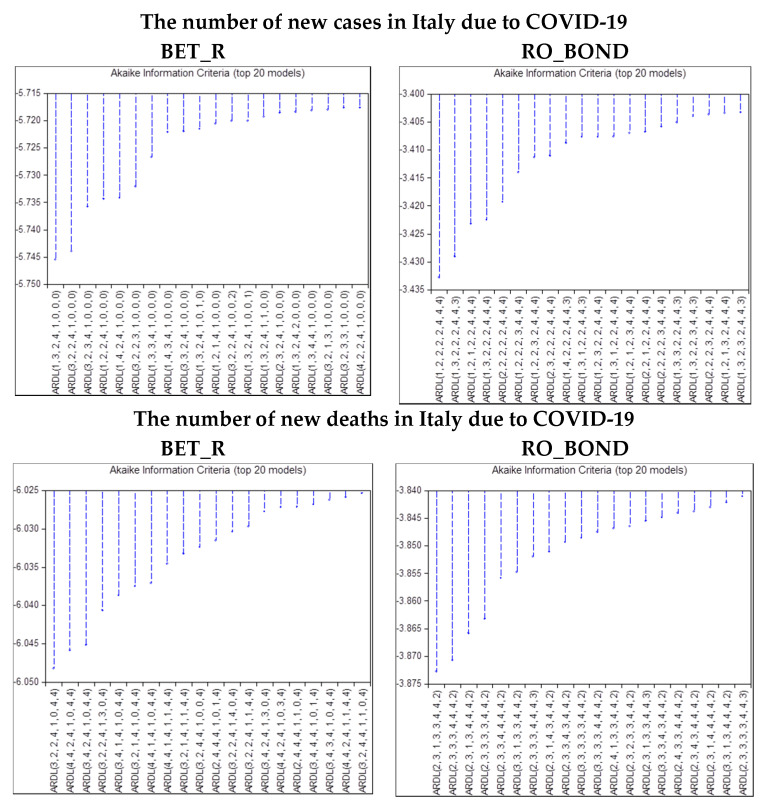
Optimal lags for the model Romania and COVID-19 (Italy). Source: authors’ own work. Notes: for the definition of variables, please see Table 1.

Figure 7 shows the results of criteria graph for the ARDL model that takes into account the number of new cases and new deaths in Italy, both for the BET stock index return and for the Romanian Government bond (10Y). Likewise, in case of Italy, in total, 1,562,500 ARDL model specifications were considered for each of the four cases.

Table 6 exhibits the selected lags for the model Romania and COVID-19 (Italy) in line with criteria graph out of Figure 7.

The results reported in Table 7 and Table 8 provides the ARDL bound test for cointegration. If the F-statistic is greater than the upper bound, then the variables comprised in the model are cointegrated and a long-run relationship befall. With reference to new cases in China models (see Table 7), the F-statistic for BET_R (18.06988) and RO_BOND (4.523219) models is greater than the upper bound of bounds value at 5%, which is suggesting that long-run relationship occur between the variables. The same result is achieved in the case of new deaths in China models, where the value of the F-Statistic is greater than the upper bound critical value. Hence, the null hypothesis is rejected, meaning that the variables in the model are cointegrated.

Regarding Italy, in all four estimated ARDL models the existence of cointegration is confirmed (see Table 8) since the F-statistic is significantly higher than the critical values in I(0) and I(1). Consequently, the examined variables are cointegrated and will move together in long-run.

Further, we will analyze the results of the long-term linkages between selected measures. Table 9 shows the outcomes regarding the long-run causal connections among variables for the model Romania and COVID-19 (China)—new cases. The short-run estimates of ARDL approach are presented in Table S1. In the first model, the number of new infection cases from China have no effect on the BET index return. However, a decrease of crude oil price leads to a higher uncertainty, consistent with Salisu, Ebuh and Usman [23], suggesting the necessity for policymakers to diminish fears in financial markets. In addition, the exchange rate negatively influences stock market return in the long-run. The Philadelphia Gold/Silver Index coefficient is positive and significant at the 5% level of significance. Hence, the coefficient of XAU_R indicates that an increase of one unit in Philadelphia Gold/Silver Index leads to over 0.2983 units increase in BET index return in the long-run. The error correction term or adjustment speed provides evidence regarding the rate of convergence to equilibrium, being highly statistically significant. The adjustment speed of −1.017783 shows that deviations from the long-term equilibrium in BET index return are corrected the following day by approximately 101.7783 percent. However, the short-run results show no impact of new infection cases of COVID-19 from China on the BET index.

Regarding the second model from Table 9, similar to the first model, the new infection cases from China does not influence Romania 10-year bond yield in the long-run. Unlike the previous model, the RO_BOND is negatively affected by XAU_R and indicates that an increase of one unit in Philadelphia Gold/Silver Index leads to over 0.3718 units decrease in RO_BOND return in the long-term. Besides, in the long-run, the return of stock market index SSE 100 negatively influences Romania 10-year bond yield. The coefficient of the error correction term is highly statistically significant. Hence, the Romanian 10-year bond will reach equilibrium with a speed of 185.3068 percent in next day. As well, the short-run results strengthen the lack of impact regarding new infection cases of COVID-19 from China on RO_BOND.

Table 10 reveals the outcomes of the long-term connection amongst variables for the model Romania and COVID-19 (China)—new deaths. The short-run results are shown in Table S2.

The empirical findings reveal that the impact is stronger in this case as compared to the model that depends on the number of new cases in China due to COVID-19 (see Table 9). However, both models shows that the number of new deaths in China due to COVID-19 has no influence on the BET index return, respectively, on the Romania 10-year bond yield, neither in the short-term, nor in the long-term. Therefore, both research hypotheses are rejected for Chinese COVID-19 figures, similar Topcu and Gulal [86] which established that emerging European countries experienced the lowest influence of the outbreak.

Table 11 and Table 12 reveals the results of serial correlation and heteroscedasticity tests for the models Romania and COVID-19 (China)—new cases and Romania and COVID-19 (China)—new deaths. The results support that the models are free from autocorrelation and heteroscedasticity.

In the case of models that take into account the effects of new cases and new deaths in Italy, unique relationships are identified between the selected variables, as opposed to the models that explored the impact of coronavirus from China. Table 13 exhibits the outcomes of the long-term causal associations between variables for the model Romania and COVID-19 (Italy)—new cases. The short-run outcomes are exhibited in Table S3. In the long-run, the results of the first model show the lack of any effect from the number of new cases of COVID-19 in Italy on BET index return. In contrast, the return of Milan stock market index FTSE MIB has a positive long-term impact on the BET index return. As well, the short-run results reveal no impact of new infection cases of COVID-19 from Italy on the BET index return. In contrast to COVID-19 figures from China, in case of Italian new cases of coronavirus, the first hypothesis is still rejected, but the second hypothesis is confirmed.

Moreover, in the second model, several statistically significant relationships are identified. There is found a positive impact of the number of new cases in Italy on the Romania 10-year bond yield in the long-term. In addition, a natural gas futures contract has a positive effect on RO_BOND, while the WTI Oil and Philadelphia Gold/Silver Index has a negative impact in the long-run. Another outstanding outcome is that new infection cases of COVID-19 from Italy negatively influence RO_BOND in the short-run, consistent with Sène, Mbengue and Allaya [36]. Therefore, the related uncertainty triggered by the health emergency may determine investors to get rid of their securities.

Table 14 exposes the findings towards long-run linkages between variables for models related to Romania and COVID-19 (Italy)—new deaths. The results of short-run estimates are presented in Table S4.

The first model out of Table 14 exhibits that the number of new deaths from Italy have no effect on the BET index return in the long-run. The Philadelphia Gold/Silver Index coefficient is positive and significant at the 5% level of significance. Hence, the coefficient value of XAU_R indicates that an increase of one unit in Philadelphia Gold/Silver Index leads to over 0.1574 units increase in BET index return in the long-term. However, the short-run results show a negative impact of new deaths cases of COVID-19 from Italy on the BET index return, in line with Okorie and Lin [58] which underlined a transitory contagion effect in the stock markets due to novel coronavirus. In addition, Erdem [55] claimed that the index returns decline and volatilities rise due to corona crisis. Hence, the first hypothesis is confirmed.

The second model shows a negative effect of the new deaths’ cases from Italy on the Romania 10-year bond yield in the long-run. In addition, the Philadelphia Gold/Silver Index and the OK crude oil future contract negatively influence RO_BOND in the long-term. Besides, in the long-run, the returns of the stock market index FTSE MIB has no impact on the 10-year Romanian bond. Nevertheless, in the short-run, results show a negative impact of new deaths cases of COVID-19 from Italy on the RO_BOND. Therefore, the second hypothesis is established.

Table 15 and Table 16 exhibit the outcomes of Breusch—Godfrey Serial correlation LM test and Breusch–Pagan–Godfrey heteroscedasticity test for the models Romania and COVID-19 (Italy)—new cases and Romania and COVID-19 (Italy)—new deaths. Hence, the models are not threatened by autocorrelation and heteroscedasticity.

### 4.3. Causality Investigation

With the purpose of exploring the causality between included variables, the Granger causality test is employed. In order to be able to apply the Granger causality test, the data series must be stationary and therefore they were turned it into stationary series. Table 17 displays the results of Granger causality test for the stock market returns and COVID-19 measures. There were identified some bidirectional causal relations between BET_R and FTMIB_R (1st lag), as well as among BET_R and IBEX35_R (1st lag). Besides, some unidirectional causal relations arise from FTSE_R (1st lag), DJIA_R (1st lag and 3rd lag), SSE100_R (1st lag, 2nd lag, and 3rd lag), and XAU_R (1st lag, 2nd lag, and 3rd lag) to BET_R. Nevertheless, no relationship was found between BET_R and the COVID-19 variables.

Table 18 shows the outcomes of causalities for the variables concerning commodities, currencies, governmental bonds, and COVID-19. The causalities for the whole world stock indexes, commodities, currencies, and COVID-19 variables are reported in Table S5. Some bidirectional relationships were found merely for the 1st lag between the 10-year Romanian bond and few stock market indices returns, namely CAC40, DAX, and IBEX 35. Besides, unidirectional relationships for 1st lag, 2nd lag, and 3rd lag occurred from returns of DJIA, S&P 500, FTSE 100, FTSE MIB, SSE 100, and the number of new cases in Italy due to COVID-19 to the 10-year Romanian bond.

## 5. Conclusions

One of the most severe stock market crashes was registered in March 2020 [79] due to the occurrence of the novel coronavirus COVID-19 pandemic [55]. The research contributions are twofold. First, we investigated whether the Romanian stock market is affected by the COVID-19 pandemic outbreak. Second, our paper explored the causalities among COVID-19 and major stock market returns, as well as between pandemic measures and several commodities. In this regard, we used daily stock market returns over the period December 31, 2019–April 20, 2020 for the following economies: USA, Spain, Italy, France, Germany, UK, China, and Romania. We have selected a wide range of variables that allow us to achieve our goal, such as stock market indices, new number of cases of illness, new number of deaths in China and Italy, exchange rate, commodity indices, Romanian bonds. As far as we know, this is the first study addressing the impact of the COVID-19 from both China and Italy crisis on the Romanian capital market and the 10-year Romanian bond.

After examining the stationarity of the selected data series and due to the mixed results, we conclude that the ARDL model is the most appropriate to explore the short-term and long-term causal associations among Romanian stock market and novel coronavirus. In the case of the model that includes the number of new deaths in China due to COVID-19, it is found that the impact of the coefficients is stronger compared to the model that depends on the number of new cases in China due to COVID-19. At the level of these two models, no effect was identified from the number of new deaths in China due to COVID-19 on the BET index return, respectively on the Romania 10-year bond yield, neither in the short-term, nor in the long-term.

With reference to the model that cover the new cases of coronavirus from Italy, short-run results provide support for a negative impact of new Italian COVID-19 cases on the Romania 10-year bond yield. Taking into account the number of new deaths in Italy we found that it has no effect on the BET index in the long-term, but the short-run results exposes a negative effect. Besides, the ARDL models showed a negative effect of the new deaths’ cases from Italy on the Romania 10-year bond yield both in the long-run and short-run.

Granger causality test exhibits bidirectional causal relations between returns of BET and FTSE MIB, IBEX, as well as a unidirectional causal relation from FTSE 100, DJIA, SSE 100, and Philadelphia Gold/Silver Index to BET index return. However, no relationship was found between the BET index return and the COVID-19 variables. Some bidirectional relationships were found between the 10-year Romanian bond and a few stock market indices (CAC 40, DAX, and IBEX 35). Unidirectional relationships occurred from returns of DJIA, S&P 500, FTSE 100, FTSE MIB, SSE100, and the number of new cases in Italy due to COVID-19 to the 10-year Romanian bond. 

Therefore, the empirical findings from ARDL model and Granger causality test confirmed both the presence of a long-term and short-term relationship between Romanian capital market and COVID-19 variables. The findings show that the Chinese COVID-19 numbers have no impact on the Romanian financial market. In addition, it was found that the 10-year Romanian bond is more sensitive to the news related to COVID-19 than the index of the Bucharest Stock Exchange, similar to Pavlyshenko [39], Mamaysky [88].

The paper may have some policy implications. As long as the BET index is not influenced by COVID-19 variables, this may suggest evidence of an inefficient market, in line with Beck, Flynn and Homanen [52], Mensi, Sensoy, Vo and Kang [72]. There are required policies to increase market efficiency though longstanding and sustainable growth rather than administering short-term interest rates [73]. The investors should seek long-term horizons of investing since the monetary and fiscal policies set by governments will alleviate the harmful effects of COVID-19. The policymakers should be aware that corona crisis may be an occasion to improve the discrepancy among Romania and developed nations of European Union. In this regard, a substantial share of the budget should be expended to alleviate this pandemic [59]. A suitable clinical stream is vital so as to ensure a reliable supervision of patients [92]. Rearrangement of public expenditure to enlarge the absorptive volume of healthcare organizations is essential [46]. Therefore, public health expenditures should be increased, along with offering direct income funding to exposed populations via cash transfers, support to affected manufacturing areas and corporations through transient tax cuts, deferral on debt reimbursements, and interim credit lines [3].

## Figures and Tables

**Table 1:** Variable descriptions.

Variables	Description	Source
**Variables towards COVID-19 pandemic outbreak**		
NC_CH	The number of new cases due to COVID-19 in China	Our World in Data
ND_CH	The number of new deaths due to COVID-19 in China	Our World in Data
NC_IT	The number of new cases due to COVID-19 in Italy	Our World in Data
ND_IT	The number of new deaths due to COVID-19 in Italy	Our World in Data
**Variables concerning stock market returns**		
DJIA_R	The daily percentage change of close price of Dow Jones Industrial Average (USA)	Thomson Reuters Eikon
SPX_R	The daily percentage change of close price of S&P 500 (USA). The S&P 500 is usually viewed as the best single gauge of large-cap U.S. equities. The index consist of 500 leading corporations and covers about 80% of existing market capitalization	Thomson Reuters Eikon
IBEX35_R	The daily percentage change of close price of IBEX 35 (Spain). The IBEX 35 index is intended to denote real-time progress of the most liquid stocks in the Spanish Stock Exchange and for use as an underlying index for trading in financial derivatives. It is composed of the 35 securities listed on the Stock Exchange	Thomson Reuters Eikon
FTMIB_R	The daily percentage change of close price of FTSE MIB (Italy). The FTSE MIB is the benchmark index for the Borsa Italiana, the Italian National Stock Exchange and covers the 40 most-traded stock classes on the exchange	Thomson Reuters Eikon
FCHI_R	The daily percentage change of close price of CAC 40 (France). The CAC 40 is a benchmark French stock market index. The index represents a capitalization-weighted measure of the 40 most significant stocks among the 100 largest market caps on the Euronext Paris (formerly the Paris Bourse)	Thomson Reuters Eikon
GDAXI_R	The daily percentage change of close price of DAX 30 (Germany). The DAX is a blue-chip stock market index comprising the 30 major German corporations trading on the Frankfurt Stock Exchange	Thomson Reuters Eikon
FTSE_R	The daily percentage change of close price of FTSE 100 (UK). The Financial Times Stock Exchange 100 Index is a share index of the 100 corporations listed on the London Stock Exchange with the highest market capitalization	Thomson Reuters Eikon
SSE100_R	The daily percentage change of close price of SSE 100 (China). SSE 100 Index consists of 100 stocks with features of most rapid operating income growth rate and highest return on equity within the universe of SSE 380 Index, and aims to reflect the overall performance of core stocks in the emerging blue chip sector that trade in Shanghai market	Thomson Reuters Eikon
BET_R	The daily percentage change of close price of BET (Romania). Bucharest Exchange Trading Index (BET) is a capitalization weighted index, comprised of the 10 most liquid stocks listed on the BSE tier 1	Thomson Reuters Eikon
**Variables regarding commodities**		
CRUDE_OIL	Cushing, OK Crude Oil Future Contract 1 (Dollars per Barrel)	Energy Information Administration
WTI	Cushing, OK WTI Spot Price FOB (Dollars per Barrel)	Energy Information Administration
NATURAL_GAS	Natural Gas Futures Contract 1 (Dollars per Million Btu)	Energy Information Administration
LSCO	The New York Mercantile Exchange (NYMEX) Light Sweet Crude Oil (WTI)	Thomson Reuters Eikon
XAU_R	The daily percentage change of close price of Philadelphia Gold/Silver Index	Thomson Reuters Eikon
**Variables regarding currencies**		
EUR_CNY	The daily percentage change of EUR/CNY	Investing.com
**Variables regarding 10-Year Government Bond Spreads**		
RO_BOND	The daily percentage change of the Romanian 10-year bond yield	Investing.com

Source: Authors’ own work.

**Table 2:** Descriptive statistics of the variables.

Variables	Mean	Median	Standard Deviation	Skewness	Kurtosis	Jarque–Bera	Probability
NC_CH	887.5000	98.5000	2040.816	5.02	34.31	3243.22	0.00
ND_CH	33.0278	10.5000	47.9956	2.13	8.31	138.78	0.00
NC_IT	1521.139	95.0000	1934.999	0.78	1.99	10.45	0.01
ND_IT	208.0139	4.5000	279.6809	0.85	2.06	11.23	0.00
DJIA_R	−0.002321	0.0000	0.0371	−0.39	5.89	2.88	0.00
SPX_R	−0.0024	0.0001	0.0341	−0.67	5.76	28.26	0.00
IBEX35_R	−0.0052	−0.0008	0.0301	−1.69	10.65	210.03	0.00
FTMIB_R	−0.0052	0.0013	0.0337	−2.53	14.67	485.29	0.00
FCHI_R	−0.0038	0.0003	0.0299	−1.16	7.51	77.15	0.00
GDAXI_R	−0.0029	0.0001	0.0299	−0.83	8.67	104.56	0.00
FTSE_R	−0.0035	0.0000	0.0260	−0.93	8.62	105.12	0.00
SSE100_R	0.0000	0.0003	0.0190	−1.65	8.49	123.27	0.00
BET_R	−0.0031	−0.0007	0.0250	−0.96	6.58	49.60	0.00
CRUDE_OIL	40.9738	49.1500	17.6997	−1.35	6.37	56.07	0.00
WTI	40.9296	49.1300	17.8440	−1.33	6.05	49.03	0.00
NATURAL_GAS	1.8352	1.8270	0.1604	0.57	2.88	4.01	0.13
LSCO	41.0201	48.1050	15.5022	−0.43	1.69	7.41	0.02
XAU_R	0.0041	0.0040	0.0455	−0.25	5.61	21.14	0.00
EUR_CNY	−0.0002	0.0000	0.0058	0.06	4.17	4.13	0.13
RO_BOND	0.0013	0.0000	0.0535	−1.52	15.66	508.19	0.00

Source: authors’ own calculations. Notes: for the definition of variables, please see Table 1.

**Table 3:** Correlation matrix.

**Variables**	**NC_CH**	**ND_CH**	**NC_IT**	**ND_IT**	**DJIA_R**	**SPX_R**	**IBEX35_R**	**FTMIB_R**	**FCHI_R**	**GDAXI_R**
NC_CH	1.0000									
ND_CH	0.7347	1.0000								
NC_IT	−0.3117	−0.4345	1.0000							
ND_IT	−0.2954	−0.4332	0.9425	1.0000						
DJIA_R	0.0232	−0.0618	0.0900	0.0822	1.0000					
SPX_R	0.0311	−0.0606	0.0908	0.0807	0.9942	1.0000				
IBEX35_R	0.0906	−0.0223	0.0646	0.0726	0.7555	0.7530	1.0000			
FTMIB_R	0.0892	−0.0314	0.0702	0.0977	0.7122	0.7113	0.8734	1.0000		
FCHI_R	0.0623	−0.0466	0.1109	0.1300	0.7406	0.7261	0.8585	0.9100	1.0000	
GDAXI_R	0.0616	−0.0623	0.1343	0.1639	0.7313	0.7165	0.8419	0.9095	0.9740	1.0000
FTSE_R	0.0129	−0.0687	0.1094	0.1318	0.7864	0.7776	0.9130	0.8539	0.8994	0.8880
SSE100_R	−0.0054	0.0591	−0.0128	0.0203	0.3293	0.3124	0.3615	0.3055	0.3959	0.3793
BET_R	0.0839	−0.0117	0.0697	0.0743	0.7429	0.7346	0.7759	0.6505	0.7256	0.7308
CRUDE_OIL	0.2257	0.3237	−0.8135	−0.8392	−0.0701	−0.0799	0.0210	−0.0508	−0.0674	−0.0863
WTI	0.2257	0.3266	−0.8278	−0.8529	−0.0852	−0.0954	0.0039	−0.0685	−0.0903	−0.1114
NATURAL_GAS	0.0176	0.0215	−0.6981	−0.6758	0.0533	0.0569	0.0347	0.0382	0.0286	0.0160
LSCO	0.2691	0.3657	−0.8894	−0.8932	−0.0013	−0.0085	0.0379	0.0202	0.0023	−0.0199
XAU_R	0.0164	0.0147	0.1509	0.1904	0.4163	0.3999	0.4578	0.3668	0.4591	0.5018
EUR_CNY	−0.0433	0.0326	−0.0121	−0.0208	−0.3536	−0.3785	−0.2787	−0.3529	−0.3018	−0.3107
RO_BOND	−0.0966	−0.0654	−0.0054	−0.0517	−0.0705	−0.0268	−0.1075	−0.1446	−0.2031	−0.1371
**Variables**	**FTSE_R**	**SSE100_R**	**BET_R**	**CRUDE_OIL**	**WTI**	**NATURAL_GAS**	**LSCO**	**XAU_R**	**EUR_CNY**	**RO_BOND**
FTSE_R	1.0000									
SSE100_R	0.3919	1.0000								
BET_R	0.7797	0.5072	1.0000							
CRUDE_OIL	−0.1034	−0.0060	−0.0341	1.0000						
WTI	−0.1252	−0.0231	−0.0552	0.9953	1.0000					
NATURAL_GAS	0.0726	0.0763	0.0807	0.6400	0.6439	1.0000				
LSCO	−0.0307	0.0508	0.0520	0.9431	0.9431	0.7375	1.0000			
XAU_R	0.5626	0.2167	0.4947	−0.1737	−0.1705	−0.0435	−0.0922	1.0000		
EUR_CNY	−0.2887	−0.1657	−0.2814	0.0790	0.0692	−0.1192	0.0006	−0.1494	1.0000	
RO_BOND	−0.1471	−0.1831	−0.0734	−0.0311	−0.0289	−0.0089	−0.0442	−0.0899	−0.3658	1.0000

Source: authors’ own calculations. Notes: for the definition of variables, please see Table 1.

**Table 4:** The outcomes of the augmented Dickey–Fuller test.

Variable	Level	1st Difference	Integration Order
	Prob.*	Prob.*	
NC_CH	0.016	0	I(0)
ND_CH	0.6591	0.0001	I(1)
NC_IT	0.7764	0	I(1)
ND_IT	0.7121	0.0265	I(1)
DJIA_R	0.0867	0	I(1)
SPX_R	0.4132	0.0001	I(1)
IBEX35_R	0.1097	0.0001	I(1)
FTMIB_R	0.0738	0.0001	I(1)
FCHI_R	0.0719	0	I(1)
GDAXI_R	0.3611	0.0001	I(1)
FTSE_R	0.3798	0.0001	I(1)
SSE100_R	0.0301	0.0001	I(0)
BET_R	0.0865	0.0001	I(1)
CRUDE_OIL	0.9977	0.0001	I(1)
WTI	0.9963	0.0001	I(1)
NATURAL_GAS	0.2127	0	I(1)
LSCO	0.9689	0	I(1)
XAU_R	0	0	I(0)
EUR_CNY	0	0	I(0)
RO_BOND	0.0003	0	I(0)

Source: authors’ own calculations. Notes: null hypothesis: has a unit root. * MacKinnon (1996) one-sided *p*-values. For the definition of variables, please see Table 1.

**Table 5:** Results of autoregressive distributed lags (ARDLs) for the model Romania and COVID-19 (China).

**ARDL—The Number of New Cases in China due to COVID-19**	
BET_R	ARDL(1, 0, 2, 1, 4, 1, 1, 2, 0)
RO_BOND	ARDL(3, 2, 3, 2, 2, 1, 4, 4, 0)
**ARDL—The Number of New Deaths in China due to COVID-19**	
BET_R	ARDL(1, 0, 2, 1, 4, 1, 1, 2, 0)
RO_BOND	ARDL(2, 2, 3, 0, 2, 2, 4, 4, 0)

Source: authors’ own calculations. Notes: for the definition of variables, please see Table 1.

**Table 6:** Results of ARDL lags for the model: Romania and COVID-19 (Italy).

**ARDL—The number of new cases in Italy due to COVID-19**	
BET_R	ARDL(1, 3, 2, 4, 1, 0, 0, 0)
RO_BOND	ARDL(1, 2, 2, 2, 2, 4, 4, 4)
**ARDL—The number of new deaths in Italy due to COVID-19**	
BET_R	ARDL(3, 2, 2, 4, 1, 0, 4, 4)
RO_BOND	ARDL(2, 3, 1, 3, 3, 4, 4, 2)

Source: authors’ own calculations. Notes: for the definition of variables, please see Table 1.

**Table 7:** The results of the ARDL bounds test for the model Romania and COVID-19 (China).

**Null Hypothesis: No Long-Run Relationships Exist**	***F*-Statistic**	
**The number of new cases in China due to COVID-19**		
BET_R	18.06988	
RO_BOND	4.523219	
**The number of new deaths in China due to COVID-19**		
BET_R	18.40808	
RO_BOND	5.358775	
**Critical Value Bounds**		
**Significance**	**I0 Bound**	**I1 Bound**
10%	1.95	3.06
5%	2.22	3.39
2.50%	2.48	3.7
1%	2.79	4.1

Source: authors’ own calculations. Notes: for the definition of variables, please see Table 1.

**Table 8:** The results of the ARDL bounds test for the model Romania and COVID-19 (Italy).

**Null Hypothesis: No Long-Run Relationships Exist**	***F*-Statistic**	
**The number of new cases in Italy due to COVID-19**		
BET_R	21.68051	
RO_BOND	7.294209	
**The number of new deaths in Italy due to COVID-19**		
BET_R	18.94637	
RO_BOND	5.32708	
**Critical Value Bounds**		
**Significance**	**I0 Bound**	**I1 Bound**
10%	2.03	3.13
5%	2.32	3.5
2.50%	2.6	3.84
1%	2.96	4.26

Source: authors’ own calculations. Notes: for the definition of variables, please see Table 1.

**Table 9:** ARDL long-run coefficients estimates for the model Romania and COVID-19 (China)—new cases.

ARDL—The Number of New Cases in China due to COVID-19					
**BET_R**					
**Variables**	**Coefficient**	**Std. Error**	***t*-Statistic**	**Prob.**	**CointEq (−1)**
SSE100_R	0.1616	0.1043	1.5489	0.1275	−1.017783(0)
EUR_CNY	−1.3775	0.6322	−2.1790	0.0339	
LSCO	−0.0016	0.0009	−1.6941	0.0962	
XAU_R	0.2983	0.0956	3.1188	0.0030	
NATURAL_GAS	−0.0022	0.0203	−0.1062	0.9159	
CRUDE_OIL	0.0068	0.0020	3.3857	0.0014	
WTI	−0.0050	0.0015	−3.3472	0.0015	
NC_CH	0.0000	0.0000	0.5168	0.6075	
C	−0.0110	0.0292	−0.3753	0.7090	
**RO_BOND**					
**Variables**	**Coefficient**	**Std. Error**	***t*-Statistic**	**Prob.**	**CointEq (−1)**
SSE100_R	−0.73407	0.317581	−2.31143	0.0257	−1.853068 (0)
EUR_CNY	−3.33276	1.262391	−2.64004	0.0115	
LSCO	0.000428	0.001982	0.21588	0.8301	
XAU_R	−0.3718	0.140512	−2.64602	0.0113	
NATURAL_GAS	−0.0295	0.034367	−0.85833	0.3955	
CRUDE_OIL	−0.00673	0.00448	−1.50213	0.1404	
WTI	0.006189	0.003557	1.74007	0.089	
NC_CH	−2E-06	0.000001	−1.22238	0.2282	
C	0.061438	0.050715	1.21143	0.2323	

Source: authors’ own calculations. Notes: for the definition of variables, please see Table 1.

**Table 10:** ARDL long-run coefficients estimates for the model Romania and COVID-19 (China)—new deaths.

ARDL—The number of new deaths in China due to COVID-19					
**BET_R**					
**Variables**	**Coefficient**	**Std. Error**	***t*-Statistic**	**Prob.**	**CointEq (−1)**
SSE100_R	0.161344	0.103218	1.563134	0.1241	−1.022253 (0)
EUR_CNY	−1.40622	0.619485	−2.26998	0.0274	
LSCO	−0.00116	0.000982	−1.18237	0.2424	
XAU_R	0.307503	0.094295	3.261086	0.002	
NATURAL_GAS	−0.01098	0.020597	−0.53307	0.5963	
CRUDE_OIL	0.00646	0.002033	3.176981	0.0025	
WTI	−0.0049	0.00148	−3.31281	0.0017	
ND_CH	−3.5E-05	0.000041	−0.8348	0.4077	
C	0.000795	0.029663	0.026797	0.9787	
**RO_BOND**					
**Variables**	**Coefficient**	**Std. Error**	***t*-Statistic**	**Prob.**	**CointEq (−1)**
SSE100_R	−0.8325	0.375288	−2.21829	0.0316	−1.578551 (0)
EUR_CNY	−2.29762	1.480246	−1.55219	0.1276	
LSCO	−0.00106	0.001518	−0.69786	0.4889	
XAU_R	−0.46095	0.162187	−2.84208	0.0067	
NATURAL_GAS	0.007984	0.045281	0.176315	0.8608	
CRUDE_OIL	−0.00652	0.005282	−1.23372	0.2237	
WTI	0.006963	0.004186	1.663637	0.1031	
ND_CH	0.000009	0.000084	0.103675	0.9179	
C	0.014044	0.066547	0.211036	0.8338	

Source: authors’ own calculations. Notes: for the definition of variables, please see Table 1.

**Table 11:** Breusch–Godfrey serial correlation Lagrange multiplier (LM) test for the model Romania and COVID-19 (China)—new cases and new deaths.

Breusch–Godfrey Serial Correlation LM Test			
**ARDL—The number of new cases in China due to COVID-19**			
**BET_R**			
*F*-statistic	1.3637	Prob. *F*(2,50)	0.2651
Obs*R-squared	3.77603	Prob. Chi-Square(2)	0.1514
**RO_BOND**			
*F*-statistic	1.551194	Prob. *F*(2,41)	0.2242
Obs*R-squared	5.135193	Prob. Chi-Square(2)	0.0767
**ARDL—The number of new deaths in China due to COVID-19**			
**BET_R**			
*F*-statistic	0.752052	Prob. *F*(2,50)	0.4767
Obs*R-squared	2.131861	Prob. Chi-Square(2)	0.3444
**RO_BOND**			
*F*-statistic	2.743942	Prob. *F*(2,43)	0.0756
Obs*R-squared	8.262179	Prob. Chi-Square(2)	0.0161

Source: authors’ own calculations. Notes: The Obs*R-squared statistic is the Breusch-Godfrey LM test statistic. This LM statistic is computed as the number of observations, times the (uncentered) R-squared from the test regression. For the definition of variables, please see Table 1.

**Table 12:** Heteroscedasticity test: Breusch–Pagan–Godfrey for the model Romania and COVID-19 (China)—new cases and new deaths.

Heteroscedasticity Test: Breusch–Pagan–Godfrey			
**ARDL—The number of new cases in China due to COVID-19**			
**BET_R**			
*F*-statistic	1.998167	Prob. *F*(20,52)	0.0237
Obs*R-squared	31.72268	Prob. Chi-Square(20)	0.0463
**RO_BOND**			
*F*-statistic	1.088975	Prob. *F*(29,43)	0.3929
Obs*R-squared	30.91112	Prob. Chi-Square(29)	0.3696
**ARDL—The number of new deaths in China due to COVID-19**			
**BET_R**			
*F*-statistic	1.228936	Prob. *F*(20,52)	0.2699
Obs*R-squared	23.43009	Prob. Chi-Square(20)	0.2682
**RO_BOND**			
*F*-statistic	1.062309	Prob. *F*(27,45)	0.4193
Obs*R-squared	28.41672	Prob. Chi-Square(27)	0.3897

Source: authors’ own calculations. Notes: The Obs*R-squared statistic for the Breusch-Pagan-Godfrey test is computed by multiplying the sample size by the coefficient of determination of the regression of squared residuals from the original regression. For the definition of variables, please see Table 1.

**Table 13:** ARDL long-run coefficients estimates for model Romania and COVID-19 (Italy)—new cases.

ARDL—The Number of New Cases in Italy due to COVID-19					
**BET_R**					
**Variables**	**Coefficient**	**Std. Error**	***t*-Statistic**	**Prob.**	**CointEq (−1)**
FTMIB_R	0.2859	0.1377	2.0760	0.0427	−0.954393 (0)
LSCO	−0.0003	0.0006	−0.4545	0.6513	
XAU_R	0.1963	0.1074	1.8279	0.0731	
NATURAL_GAS	0.0123	0.0163	0.7532	0.4546	
CRUDE_OIL	0.0024	0.0013	1.8294	0.0729	
WTI	−0.0021	0.0012	−1.7002	0.0948	
NC_IT	0.0000	0.0000	0.0103	0.9918	
C	−0.0256	0.0295	−0.8669	0.3898	
**RO_BOND**					
**Variables**	**Coefficient**	**Std. Error**	***t*-Statistic**	**Prob.**	**CointEq (−1)**
FTMIB_R	0.5133	0.3556	1.4437	0.1559	−1.147405 (0)
LSCO	−0.0068	0.0041	−1.6445	0.1072	
XAU_R	−0.7336	0.2267	−3.2362	0.0023	
NATURAL_GAS	0.1743	0.0593	2.9375	0.0052	
CRUDE_OIL	0.0185	0.0087	2.1270	0.0391	
WTI	−0.0187	0.0073	−2.5465	0.0145	
NC_IT	0.0000	0.0000	−3.0230	0.0042	
C	0.0342	0.0866	0.3944	0.6952	

Source: authors’ own calculations. Notes: for the definition of variables, please see Table 1.

**Table 14:** ARDL long-run coefficients estimates for model Romania and COVID-19 (Italy)—new deaths.

ARDL—The Number of New Deaths in Italy due to COVID-19					
**BET_R**					
**Variables**	**Coefficient**	**Std. Error**	***t*-Statistic**	**Prob.**	**CointEq (−1)**
FTMIB_R	0.3143	0.0643	4.8907	0.0000	−1.647813 (0)
LSCO	−0.0009	0.0005	−1.6594	0.1040	
XAU_R	0.1574	0.0662	2.3773	0.0218	
NATURAL_GAS	−0.0108	0.0107	−1.0016	0.3219	
CRUDE_OIL	0.0027	0.0008	3.4207	0.0013	
WTI	−0.0013	0.0007	−1.8479	0.0712	
ND_IT	0.0000	0.0000	1.3777	0.1751	
C	−0.0045	0.0153	−0.2954	0.7691	
**RO_BOND**					
**Variables**	**Coefficient**	**Std. Error**	***t*-Statistic**	**Prob.**	**CointEq (−1)**
FTMIB_R	0.1323	0.3058	0.4327	0.6674	−1.204853(0)
LSCO	−0.0105	0.0029	−3.6061	0.0008	
XAU_R	−0.5498	0.2305	−2.3852	0.0216	
NATURAL_GAS	0.1286	0.0571	2.2515	0.0295	
CRUDE_OIL	0.0240	0.0085	2.8202	0.0072	
WTI	−0.0192	0.0076	−2.5115	0.0159	
ND_IT	−0.0002	0.0001	−2.7338	0.0091	
C	0.0504	0.0632	0.7967	0.4300	

Source: authors’ own calculations. Notes: for the definition of variables, please see Table 1.

**Table 15:** Breusch–Godfrey serial correlation LM test for the model Romania and COVID-19 (Italy)—new cases and new deaths.

Breusch–Godfrey Serial Correlation LM Test:			
**ARDL—The number of new cases in Italy due to COVID-19**			
**BET_R**			
*F*-statistic	0.636347	Prob. *F*(2,52)	0.5333
Obs*R-squared	1.743982	Prob. Chi-Square(2)	0.4181
**RO_BOND**			
*F*-statistic	1.679769	Prob. *F*(4,40)	0.1737
Obs*R-squared	10.49876	Prob. Chi-Square(4)	0.0328
**ARDL—The number of new deaths in Italy due to COVID-19**			
**BET_R**			
*F*-statistic	0.057834	Prob. *F*(2,43)	0.9439
Obs*R-squared	0.19584	Prob. Chi-Square(2)	0.9067
**RO_BOND**			
*F*-statistic	2.062798	Prob. *F*(2,41)	0.1401
Obs*R-squared	6.674006	Prob. Chi-Square(2)	0.0355

Source: authors’ own calculations. Notes: The Obs*R-squared statistic is the Breusch-Godfrey LM test statistic. This LM statistic is computed as the number of observations, times the (uncentered) R-squared from the test regression. For the definition of variables, please see Table 1.

**Table 16:** Heteroscedasticity test: Breusch–Pagan–Godfrey for the model Romania and COVID-19 (Italy)—new cases and new deaths.

Heteroscedasticity Test: Breusch–Pagan–Godfrey			
**ARDL—The number of new cases in Italy due to COVID-19**			
**BET_R**			
*F*-statistic	1.708739	Prob. *F*(18,54)	0.0665
Obs*R-squared	26.49074	Prob. Chi-Square(18)	0.0891
**RO_BOND**			
*F*-statistic	0.693446	Prob. *F*(28,44)	0.8464
Obs*R-squared	22.35071	Prob. Chi-Square(28)	0.7648
**ARDL—The number of new deaths in Italy due to COVID-19**			
**BET_R**			
*F*-statistic	0.80796	Prob. *F*(27,45)	0.7191
Obs*R-squared	23.83434	Prob. Chi-Square(27)	0.6395
**RO_BOND**			
*F*-statistic	0.626455	Prob. *F*(29,43)	0.9063
Obs*R-squared	21.68164	Prob. Chi-Square(29)	0.8331

Source: authors’ own calculations. Notes: The Obs*R-squared statistic for the Breusch-Pagan-Godfrey test is computed by multiplying the sample size by the coefficient of determination of the regression of squared residuals from the original regression. For the definition of variables, please see Table 1.

**Table 17:** The results of the Granger causality test for the stock market and COVID-19 variables.

Null Hypothesis	1st Lag		2nd Lag		3rd Lag	
	*F*-Statistic	Prob.	*F*-Statistic	Prob.	*F*-Statistic	Prob.
DFCHI_R does not Granger Cause DBET_R	2.6267	0.1095	1.37666	0.2593	1.03323	0.3837
DBET_R does not Granger Cause DFCHI_R	0.01526	0.902	0.67225	0.5139	2.73881	0.0503
DWTI does not Granger Cause DBET_R	0.32344	0.5713	0.15567	0.8561	0.89465	0.4487
DBET_R does not Granger Cause DWTI	0.66746	0.4166	0.55401	0.5772	0.60479	0.6142
DCRUDE_OIL does not Granger Cause DBET_R	1.64744	0.2034	1.19169	0.3099	1.54876	0.2102
DBET_R does not Granger Cause DCRUDE_OIL	1.40219	0.2403	0.74496	0.4785	1.09251	0.3585
DGDAXI_R does not Granger Cause DBET_R	0.54561	0.4625	1.70531	0.1893	1.15653	0.333
DBET_R does not Granger Cause DGDAXI_R	0.63702	0.4274	1.82947	0.1682	2.55856	0.0625
DDJIA_R does not Granger Cause DBET_R	0.08379	0.7731	1.01848	0.3665	1.24507	0.3005
DBET_R does not Granger Cause DDJIA_R	1.91735	0.1704	0.54163	0.5843	0.36964	0.7752
DFTSE_R does not Granger Cause DBET_R	0.14757	0.702	1.46304	0.2386	0.94017	0.4264
DBET_R does not Granger Cause DFTSE_R	0.34236	0.5603	0.82895	0.4408	0.90187	0.4451
DFTMIB_R does not Granger Cause DBET_R	3.9811	0.0498	0.68299	0.5085	2.40174	0.0755
DBET_R does not Granger Cause DFTMIB_R	2.40769	0.1251	1.63062	0.2033	1.53362	0.214
DIBEX35_R does not Granger Cause DBET_R	5.99134	0.0168	5.79833	0.0047	3.77034	0.0146
DBET_R does not Granger Cause DIBEX35_R	5.93584	0.0173	2.58061	0.083	3.46318	0.0211
DJIA_R does not Granger Cause DBET_R	4.84108	0.031	2.32679	0.1052	3.07207	0.0337
DBET_R does not Granger Cause DJIA_R	3.6263	0.0609	0.96526	0.386	0.85631	0.4683
DNATURAL_G does not Granger Cause DBET_R	2.61024	0.1105	3.06162	0.0532	2.01611	0.1202
DBET_R does not Granger Cause DNATURAL_G	4.6538	0.0343	2.93068	0.06	2.76934	0.0485
DNC_IT does not Granger Cause DBET_R	1.88151	0.1744	0.24766	0.7813	2.31234	0.0841
DBET_R does not Granger Cause DNC_IT	6.78189	0.0112	3.57262	0.0334	3.72495	0.0155
DND_CH does not Granger Cause DBET_R	0.00174	0.9668	0.00364	0.9964	0.00707	0.9992
DBET_R does not Granger Cause DND_CH	0.00076	0.9781	0.00208	0.9979	0.02642	0.9941
DND_IT does not Granger Cause DBET_R	1.14888	0.2874	0.76009	0.4715	0.49269	0.6886
DBET_R does not Granger Cause DND_IT	0.00748	0.9313	0.67359	0.5132	1.80249	0.1553
DLSCO does not Granger Cause DBET_R	0.03988	0.8423	0.9342	0.3978	0.91111	0.4405
DBET_R does not Granger Cause DLSCO	7.33898	0.0084	5.77264	0.0048	3.88014	0.0129
DSPX_R does not Granger Cause DBET_R	0.17873	0.6737	1.34967	0.2661	1.65264	0.1858
DBET_R does not Granger Cause DSPX_R	1.82924	0.1804	0.52552	0.5936	0.34511	0.7928
SSE100_R does not Granger Cause DBET_R	7.74827	0.0069	4.02162	0.0223	2.87382	0.0428
DBET_R does not Granger Cause SSE100_R	0.34946	0.5563	0.65952	0.5203	2.16779	0.1001
EUR_CNY does not Granger Cause DBET_R	0.21832	0.6417	0.61712	0.5424	0.66098	0.579
DBET_R does not Granger Cause EUR_CNY	11.4005	0.0012	4.48184	0.0148	2.86132	0.0434
NC_CH does not Granger Cause DBET_R	0.02747	0.8688	0.01495	0.9852	0.00963	0.9987
DBET_R does not Granger Cause NC_CH	0.01858	0.892	0.00141	0.9986	0.02117	0.9958
XAU_R does not Granger Cause DBET_R	8.85791	0.004	13.0642	0.00002	8.66267	0.00006
DBET_R does not Granger Cause XAU_R	17.5622	0.00008	12.3505	0.00003	9.59776	0.00002

Source: authors’ own calculations. Notes: for the definition of variables, please see Table 1.

**Table 18:** The results of the Granger causality test for commodities, currencies, governmental bonds, and COVID-19 variables.

Null Hypothesis	1st Lag		2nd Lag		3rd Lag	
	*F*-Statistic	Prob.	*F*-Statistic	Prob.	*F*-Statistic	Prob.
DFCHI_R does not Granger Cause RO_BOND	7.93244	0.0063	4.10612	0.0207	2.96656	0.0382
RO_BOND does not Granger Cause DFCHI_R	5.35818	0.0235	5.90784	0.0043	5.71237	0.0015
DWTI does not Granger Cause RO_BOND	1.40788	0.2393	2.84061	0.0652	2.52773	0.0649
RO_BOND does not Granger Cause DWTI	1.84894	0.1781	2.82801	0.066	1.84005	0.1485
DCRUDE_OIL does not Granger Cause RO_BOND	0.28071	0.5979	0.18731	0.8296	1.73016	0.1693
RO_BOND does not Granger Cause DCRUDE_OIL	2.24912	0.1381	1.54906	0.2197	0.96236	0.4158
DGDAXI_R does not Granger Cause RO_BOND	8.83453	0.004	4.36102	0.0165	3.97272	0.0115
RO_BOND does not Granger Cause DGDAXI_R	6.57828	0.0124	5.37455	0.0068	4.73576	0.0047
DDJIA_R does not Granger Cause RO_BOND	8.42463	0.0049	6.77884	0.0021	5.22182	0.0027
RO_BOND does not Granger Cause DDJIA_R	2.77374	0.1002	1.50012	0.2303	1.15489	0.3337
DFTSE_R does not Granger Cause RO_BOND	7.81722	0.0066	3.88167	0.0253	3.42563	0.0221
RO_BOND does not Granger Cause DFTSE_R	2.39641	0.126	3.53877	0.0344	3.00637	0.0365
DFTMIB_R does not Granger Cause RO_BOND	24.5669	0.000005	12.2384	0.00003	11.8882	0.000002
RO_BOND does not Granger Cause DFTMIB_R	0.03944	0.8431	0.45054	0.6391	0.92968	0.4313
DIBEX35_R does not Granger Cause RO_BOND	4.56719	0.036	2.23299	0.1149	1.50269	0.222
RO_BOND does not Granger Cause DIBEX35_R	5.16866	0.026	3.34464	0.0411	3.60425	0.0178
DJIA_R does not Granger Cause RO_BOND	19.8188	0.00003	11.5107	0.00005	7.49281	0.0002
RO_BOND does not Granger Cause DJIA_R	3.31803	0.0726	0.89821	0.4119	0.18455	0.9065
DNATURAL_GAS does not Granger Cause RO_BOND	1.33944	0.251	0.66142	0.5194	0.45155	0.7171
RO_BOND does not Granger Cause DNATURAL_GAS	0.50062	0.4815	0.43031	0.652	1.03227	0.3841
DNC_IT does not Granger Cause RO_BOND	7.62726	0.0073	4.77217	0.0115	3.05509	0.0344
RO_BOND does not Granger Cause DNC_IT	0.09051	0.7644	0.15265	0.8587	2.58859	0.0603
DND_CH does not Granger Cause RO_BOND	0.01047	0.9188	0.34077	0.7124	0.22026	0.882
RO_BOND does not Granger Cause DND_CH	0.10515	0.7467	0.02699	0.9734	0.05421	0.9832
DND_IT does not Granger Cause RO_BOND	0.16266	0.6879	2.83622	0.0655	3.69605	0.016
RO_BOND does not Granger Cause DND_IT	1.30755	0.2566	2.40189	0.0981	2.24727	0.091
DLSCO does not Granger Cause RO_BOND	2.62586	0.1095	1.35676	0.2643	0.87127	0.4605
RO_BOND does not Granger Cause DLSCO	0.04223	0.8378	0.07082	0.9317	0.28769	0.8341
DSPX_R does not Granger Cause RO_BOND	7.23441	0.0089	5.2898	0.0073	3.98772	0.0113
RO_BOND does not Granger Cause DSPX_R	1.93361	0.1686	0.73046	0.4854	0.53808	0.6578
SSE100_R does not Granger Cause RO_BOND	5.93434	0.0173	3.43564	0.0377	2.88714	0.042
RO_BOND does not Granger Cause SSE100_R	0.16848	0.6827	0.55591	0.5761	0.58164	0.6291
NC_CH does not Granger Cause RO_BOND	0.04289	0.8365	0.01927	0.9809	0.30151	0.8242
RO_BOND does not Granger Cause NC_CH	0.01696	0.8967	0.0044	0.9956	0.00846	0.9989

Source: authors’ own calculations. Notes: for the definition of variables, please see Table 1.

## Supplementary Materials

The following are available online at https://www.mdpi.com/1660-4601/17/18/6729/s1, Table S1: ARDL short-run coefficient estimates for the model Romania and COVID-19 (China)—new cases, Table S2: ARDL short-run coefficient estimates for the model Romania and COVID-19 (China)—new deaths, Table S3: ARDL short-run coefficient estimates for the model Romania and COVID-19 (Italy)—new cases, Table S4: ARDL short-run coefficient estimates for the model Romania and COVID-19 (Italy)—new deaths, Table S5: The results of the Granger causality test for world stock indexes, commodities, currencies and COVID-19 variables.
