# Supplementary material for: Stock Market Reactions to COVID-19 Pandemic Outbreak: Quantitative Evidence from ARDL Bounds Tests and Granger Causality Analysis
Source: Int J Environ Res Public Health. 2020 Sep 15;17(18):6729. doi: 10.3390/ijerph17186729 (PMC7558856; doi:10.3390/ijerph17186729)
Supplement: Supplementary file 1 [file ijerph-17-06729-s001.pdf]

Supplementary Material

Table S1. ARDL short-run coefficient estimates for the model Romania and COVID-19 (China)—new cases.

**ARDL—The Number of New Cases in China due to COVID-19**				
**BET_R**				
**Variables**	**Coefficient**	**Std. Error**	***t*-Statistic**	**Prob.**
D(SSE100_R)	0.164481	0.103782	1.584868	0.1191
D(EUR_CNY)	−0.844627	0.354823	−2.380415	0.021
D(EUR_CNY(−1))	1.170755	0.372566	3.142406	0.0028
D(LSCO)	0.002462	0.000896	2.749226	0.0082
D(XAU_R)	0.095487	0.04151	2.300351	0.0255
D(XAU_R(−1))	0.095401	0.054108	1.763156	0.0837
D(XAU_R(−2))	−0.1817	0.048485	−3.747515	0.0004
D(XAU_R(−3))	0.180338	0.043903	4.107643	0.0001
D(NATURAL_GAS)	0.101902	0.03134	3.251525	0.002
D(CUSHING_OIL)	0.002898	0.001539	1.883225	0.0653
D(WTI)	−0.002608	0.001476	−1.767505	0.083
D(WTI(−1))	−0.001469	0.000727	−2.019062	0.0487
D(NC_CH)	0	0.000001	0.516892	0.6074
**CointEq(−1)**	**−1.017783**	**0.104858**	**−9.706297**	**0**
**RO_BOND**				
**Variables**	**Coefficient**	**Std. Error**	***t*-Statistic**	**Prob.**
D(RO_BOND(−1))	0.605034	0.224319	2.697205	0.0099
D(RO_BOND(−2))	0.179495	0.142653	1.258262	0.2151
D(SSE100_R)	−0.458222	0.307938	−1.488034	0.144
D(SSE100_R(−1))	0.416106	0.300009	1.386977	0.1726
D(EUR_CNY)	−3.760744	1.162644	−3.234648	0.0023
D(EUR_CNY(−1))	1.358541	1.150085	1.181253	0.244
D(EUR_CNY(−2))	−1.293318	1.134934	−1.139554	0.2608
D(LSCO)	−0.001214	0.00289	−0.420176	0.6764
D(LSCO(−1))	−0.007986	0.004673	−1.709111	0.0946
D(XAU_R)	−0.212128	0.142993	−1.483485	0.1452
D(XAU_R(−1))	0.467853	0.156634	2.986908	0.0046
D(NATURAL_GAS)	−0.293654	0.101021	−2.906849	0.0058
D(CRUDE_OIL)	−0.005892	0.005341	−1.103109	0.2761
D(CRUDE_OIL(−1))	−0.003585	0.004966	−0.721889	0.4743
D(CRUDE_OIL(−2))	0.017481	0.005209	3.356299	0.0017
D(CRUDE_OIL(−3))	−0.009662	0.005621	−1.718914	0.0928
D(WTI)	0.006377	0.005139	1.24101	0.2213
D(WTI(−1))	−0.000379	0.004224	−0.089743	0.9289
D(WTI(−2))	−0.012309	0.004136	−2.975965	0.0048
D(WTI(−3))	0.010707	0.004126	2.595061	0.0129
D(NC_CH)	−0.000003	0.000003	−1.181846	0.2438
**CointEq(−1)**	**−1.853068**	**0.30843**	**−6.008062**	**0**

Source: authors' own calculations. Notes: for the definition of variables, please see Table 1.

Table S2. ARDL short-run coefficient estimates for the model Romania and COVID-19 (China)—new deaths.

**ARDL—The Number of New Deaths in China due to COVID-19**				
**BET_R**				
**Variables**	**Coefficient**	**Std. Error**	***t*-Statistic**	**Prob.**
D(SSE100_R)	0.164935	0.103143	1.599086	0.1159
D(EUR_CNY)	−0.89991	0.350557	−2.56708	0.0132
D(EUR_CNY(−1))	1.131745	0.374804	3.019567	0.0039
D(LSCO)	0.002525	0.000891	2.832864	0.0065
D(XAU_R)	0.096856	0.041342	2.342805	0.023
D(XAU_R(−1))	0.093231	0.053941	1.728372	0.0899
D(XAU_R(−2))	−0.18393	0.048032	−3.82925	0.0003
D(XAU_R(−3))	0.175421	0.043977	3.988903	0.0002
D(NATURAL_GAS)	0.098444	0.031355	3.139664	0.0028

D(CRUDE_OIL)	0.002658	0.001552	1.713054	0.0927
D(WTI)	−0.00247	0.001479	−1.67193	0.1005
D(WTI(−1)	−0.00138	0.000732	−1.88821	0.0646
D(ND_CH)	−3.5E-05	0.000042	−0.83322	0.4085
**CointEq(−1)**	**−1.02225**	**0.104627**	**−9.77043**	**0**
**RO_BOND**				
**Variables**	**Coefficient**	**Std. Error**	***t*-Statistic**	**Prob.**
D(RO_BOND)	0.384412	0.133015	2.889987	0.0059
D(SSE100_R)	−0.41664	0.308217	−1.35177	0.1832
D(SSE100_R(−1))	0.393341	0.299711	1.3124	0.196
D(EUR_CNY)	−3.49695	1.078339	−3.2429	0.0022
D(EUR_CNY(−1))	1.190246	1.107438	1.074774	0.2882
D(EUR_CNY(−2))	−2.17048	1.117656	−1.94199	0.0584
D(LSCO)	−0.00167	0.002393	−0.69912	0.4881
D(XAU_R)	−0.2433	0.143602	−1.69427	0.0971
D(XAU_R(−1))	0.454267	0.158975	2.857477	0.0064
D(NATURAL_GAS)	−0.19401	0.099068	−1.95839	0.0564
D(NATURAL_GAS(−1))	−0.14978	0.106777	−1.40272	0.1676
D(CRUDE_OIL)	−0.00783	0.005125	−1.52846	0.1334
D(CRUDE_OIL(−1))	−0.00749	0.004245	−1.76391	0.0845
D(CRUDE_OIL(−2))	0.019834	0.005071	3.911492	0.0003
D(CRUDE_OIL(−3))	−0.00922	0.005183	−1.77856	0.0821
D(WTI)	0.007628	0.005036	1.514727	0.1368
D(WTI(−1))	−0.00095	0.004195	−0.22659	0.8218
D(WTI(−2))	−0.01402	0.004002	−3.50288	0.0011
D(WTI(−3))	0.011578	0.003619	3.199502	0.0025
D(ND_CH)	0.000014	0.000133	0.103833	0.9178
**CointEq(−1)**	**−1.57855**	**0.200093**	**−7.88908**	**0**

Source: authors' own calculations. Notes: for the definition of variables, please see Table 1.

Table S3. ARDL short-run coefficient estimates for the model Romania and COVID-19 (Italy)—new cases.

**ARDL—The Number of New Cases in Italy due to COVID-19**				
**BET_R**				
**Variables**	**Coefficient**	**Std. Error**	***t*-Statistic**	**Prob.**
D(FTSE_MIB_R)	0.157907	0.058351	2.706136	0.0091
D(FTSE_MIB_R(−1))	−0.213515	0.059457	−3.591066	0.0007
D(FTSE_MIB_R(−2))	0.092454	0.063485	1.456327	0.1511
D(LSCO)	0.003089	0.000763	4.046168	0.0002
D(LSCO(−1))	−0.001852	0.000992	−1.866385	0.0674
D(XAU_R)	0.076655	0.041669	1.839635	0.0713
D(XAU_R(−1))	0.104701	0.049567	2.112284	0.0393
D(XAU_R(−2))	−0.074967	0.041338	−1.81353	0.0753
D(XAU_R(−3))	0.088817	0.037073	2.395729	0.0201
D(NATURAL_GAS)	0.099548	0.028511	3.491587	0.001
D(CRUDE_OIL)	0.00228	0.001177	1.937014	0.058
D(WTI)	−0.001972	0.001102	−1.788725	0.0793
D(NC_IT)	0	0.000002	0.010324	0.9918
**CointEq(−1)**	**−0.954393**	**0.127437**	**−7.489127**	**0**
**RO_BOND**				
**Variables**	**Coefficient**	**Std. Error**	***t*-Statistic**	**Prob.**
D(FTSE_MIB_R)	0.023339	0.218468	0.106832	0.9154
D(FTSE_MIB_R(−1))	−0.444661	0.205025	−2.168811	0.0355
D(LSCO)	0.00139	0.00284	0.489522	0.6269
D(LSCO(−1))	−0.00594	0.004608	−1.289191	0.2041
D(XAU_R)	−0.317075	0.15844	−2.001229	0.0516
D(XAU_R(−1))	0.466439	0.162571	2.869146	0.0063
D(NATURAL_GAS)	−0.19138	0.09544	−2.005237	0.0511
D(NATURAL_GAS(−1))	−0.270018	0.114448	−2.359303	0.0228
D(CRUDE_OIL)	0.006123	0.005008	1.222701	0.228
D(CRUDE_OIL(−1))	−0.001167	0.005122	−0.227798	0.8209
D(CRUDE_OIL(−2))	0.008228	0.006744	1.22	0.229
D(CRUDE_OIL(−3))	−0.019859	0.005687	−3.492211	0.0011
D(WTI)	−0.004427	0.004413	−1.003185	0.3213

D(WTI(−1))	−0.002618	0.004662	−0.561448	0.5773
D(WTI(−2))	−0.000775	0.004786	−0.161915	0.8721
D(WTI(−3))	0.020925	0.004174	5.013635	0
D(NC_IT)	−0.000019	0.000017	−1.088745	0.2822
D(NC_IT(−1))	−0.000021	0.000023	−0.914533	0.3654
D(NC_IT(−2))	0.00005	0.00002	2.496999	0.0163
D(NC_IT(−3))	0.000025	0.000014	1.695087	0.0971
**CointEq(−1)**	**−1.147405**	**0.124965**	**−9.181827**	**0**

Source: authors' own calculations. Notes: for the definition of variables, please see Table 1.

Table S4. ARDL short-run coefficient estimates for the model Romania and COVID-19 (Italy)—new deaths.

ARDL—The Number of New Deaths in Italy due to COVID-19				
BET_R				
**Variables**	**Coefficient**	**Std. Error**	***t*-Statistic**	**Prob.**
D(BET_R(−1))	0.532952	0.173004	3.080574	0.0035
D(BET_R(−2))	0.274404	0.097833	2.80481	0.0074
D(FTSE_MIB_R)	0.165946	0.05213	3.183305	0.0026
D(FTSE_MIB_R(−1))	−0.30499	0.064034	−4.76296	0
D(LSCO)	0.00277	0.000721	3.839906	0.0004
D(LSCO(−1))	−0.00143	0.001079	−1.32193	0.1929
D(XAU_R)	0.120443	0.04283	2.812139	0.0073
D(XAU_R(−1))	0.072502	0.050044	1.448747	0.1543
D(XAU_R(−2))	−0.08257	0.049824	−1.65722	0.1044
D(XAU_R(−3))	0.106887	0.042383	2.521909	0.0153
D(NATURAL_GAS)	0.057822	0.026244	2.20324	0.0327
D(CRUDE_OIL)	0.004447	0.001288	3.453745	0.0012
D(WTI)	−0.00357	0.001171	−3.04672	0.0039
D(WTI(−1))	0.000768	0.000925	0.829792	0.411
D(WTI(−2))	−0.00053	0.000863	−0.61551	0.5413
D(WTI(−3))	−0.00135	0.00073	−1.85595	0.07
D(ND_IT)	−2.2E-05	0.00003	−0.7062	0.4837
D(ND_IT(−1))	−2.6E-05	0.000034	−0.7619	0.4501
D(ND_IT(−2))	−7.4E-05	0.000032	−2.35702	0.0228
D(ND_IT(−3))	−0.00007	0.000026	−2.66636	0.0106
**CointEq(−1)**	**−1.64781**	**0.198051**	**−8.32017**	**0**
RO_BOND				
**Variables**	**Coefficient**	**Std. Error**	***t*-Statistic**	**Prob.**
D(RO_BOND)	0.385039	0.115268	3.340369	0.0017
D(FTSE_MIB_R)	0.118664	0.178892	0.663329	0.5107
D(FTSE_MIB_R(−1))	−0.5094	0.170639	−2.98523	0.0047
D(FTSE_MIB_R(−2))	0.604848	0.166686	3.628679	0.0008
D(LSCO)	−0.00402	0.002598	−1.54746	0.1291
D(XAU_R)	0.079132	0.126799	0.624075	0.5359
D(XAU_R(−1))	0.022838	0.143089	0.159607	0.8739
D(XAU_R(−2))	0.433998	0.138481	3.133986	0.0031
D(NATURAL_GAS)	−0.19144	0.081483	−2.34943	0.0235
D(NATURAL_GAS(−1))	0.062996	0.11798	0.533957	0.5961
D(NATURAL_GAS(−2))	−0.21711	0.082992	−2.61606	0.0122
D(CRUDE_OIL)	0.016395	0.005028	3.26053	0.0022
D(CRUDE_OIL(−1))	−0.0152	0.003644	−4.17073	0.0001
D(CRUDE_OIL(−2))	0.015609	0.004546	3.433366	0.0013
D(CRUDE_OIL(−3))	−0.02313	0.004526	−5.11018	0
D(WTI)	−0.01262	0.004727	−2.66936	0.0107
D(WTI(−1))	0.006931	0.003594	1.928216	0.0604
D(WTI(−2))	−0.00735	0.0037	−1.98495	0.0536
D(WTI(−3))	0.022317	0.003349	6.664448	0
D(ND_IT)	0.000156	0.000101	1.540406	0.1308
D(ND_IT(−1))	−0.00045	0.000113	−4.01409	0.0002
**CointEq(−1)**	**−1.20485**	**0.163461**	**−7.37088**	**0**

Source: authors' own calculations. Notes: for the definition of variables, please see Table 1.

Table S5. The results of the Granger causality test for world stock indexes, commodities, currencies and COVID-19 variables.

Null Hypothesis	1st Lag		2nd Lag		3rd Lag	
	F-Statistic	Prob.	*F*-Statistic	Prob.	*F*-Statistic	Prob.
DWTI does not Granger Cause DFCHI_R	1.0322	0.313	0.5527	0.5779	0.69504	0.5584
DFCHI_R does not Granger Cause DWTI	1.47337	0.2288	3.98796	0.023	2.61148	0.0586
DCRUDE_OIL does not Granger Cause DFCHI_R	3.49241	0.0657	1.61731	0.2059	2.73393	0.0506
DFCHI_R does not Granger Cause DCRUDE_OIL	3.24732	0.0757	4.7043	0.0122	3.235	0.0277
DGDAXI_R does not Granger Cause DFCHI_R	2.44856	0.122	1.64928	0.1997	0.76334	0.5187
DFCHI_R does not Granger Cause DGDAXI_R	5.6116	0.0205	2.90486	0.0615	1.52625	0.2159
DDJIA_R does not Granger Cause DFCHI_R	2.96338	0.0895	1.11077	0.3351	2.36803	0.0786
DFCHI_R does not Granger Cause DDJIA_R	25.1712	0.000004	5.24219	0.0076	3.56303	0.0187
DFTSE_R does not Granger Cause DFCHI_R	0.59606	0.4426	0.33488	0.7166	0.07798	0.9717
DFCHI_R does not Granger Cause DFTSE_R	6.51566	0.0128	3.52498	0.0349	2.07998	0.1113
DFTMIB_R does not Granger Cause DFCHI_R	16.5192	0.0001	5.58971	0.0056	3.70124	0.0159
DFCHI_R does not Granger Cause DFTMIB_R	4.31817	0.0413	2.91604	0.0609	1.86974	0.1433
DIBEX35_R does not Granger Cause DFCHI_R	4.20107	0.044	7.96267	0.0008	3.7264	0.0154
DFCHI_R does not Granger Cause DIBEX35_R	0.31584	0.5759	0.38985	0.6786	0.93954	0.4267
DJIA_R does not Granger Cause DFCHI_R	1.22506	0.2721	3.00503	0.0561	3.49582	0.0203
DFCHI_R does not Granger Cause DJIA_R	20.2153	0.00003	7.63484	0.001	3.97034	0.0116
DNATURAL_GAS does not Granger Cause DFCHI_R	3.99329	0.0495	5.65658	0.0053	3.43338	0.0219
DFCHI_R does not Granger Cause DNATURAL_GAS	4.23896	0.0431	2.30684	0.1072	2.62814	0.0575
DNC_IT does not Granger Cause DFCHI_R	1.39317	0.2418	0.32779	0.7216	4.08753	0.0101
DFCHI_R does not Granger Cause DNC_IT	0.59281	0.4439	0.44849	0.6404	0.29292	0.8304
DND_CH does not Granger Cause DFCHI_R	0.04532	0.832	0.02151	0.9787	0.02095	0.9958
DFCHI_R does not Granger Cause DND_CH	0.0093	0.9235	0.10122	0.9039	0.09847	0.9606
DND_IT does not Granger Cause DFCHI_R	1.74723	0.1904	3.00537	0.056	1.73521	0.1683
DFCHI_R does not Granger Cause DND_IT	3.23526	0.0763	1.28527	0.2831	5.2365	0.0026
DLSCO does not Granger Cause DFCHI_R	0.27343	0.6026	3.68697	0.0301	3.5286	0.0195
DFCHI_R does not Granger Cause DLSCO	1.04446	0.3102	0.75845	0.4723	1.97998	0.1255
DSPX_R does not Granger Cause DFCHI_R	2.21746	0.1408	1.2584	0.2905	2.13477	0.1042
DFCHI_R does not Granger Cause DSPX_R	25.3444	0.000003	4.94291	0.0099	3.44948	0.0214
SSE100_R does not Granger Cause DFCHI_R	4.30932	0.0415	3.74938	0.0285	3.32022	0.025
DFCHI_R does not Granger Cause SSE100_R	0.00163	0.9679	0.02765	0.9727	1.28404	0.2872
EUR_CNY does not Granger Cause DFCHI_R	0.65507	0.421	0.38908	0.6792	4.71085	0.0049
DFCHI_R does not Granger Cause EUR_CNY	10.8984	0.0015	4.23084	0.0185	5.54855	0.0019
NC_CH does not Granger Cause DFCHI_R	0.04487	0.8328	0.03433	0.9663	0.02894	0.9933
DFCHI_R does not Granger Cause NC_CH	0.00258	0.9596	0.10397	0.9014	0.09209	0.9642
XAU_R does not Granger Cause DFCHI_R	0.12374	0.726	4.94469	0.0098	4.34698	0.0074
DFCHI_R does not Granger Cause XAU_R	2.7382	0.1023	3.38374	0.0396	3.79388	0.0142
DCRUDE_OIL does not Granger Cause DWTI	0.04638	0.8301	0.03536	0.9653	0.1366	0.9378

DWTI does not Granger Cause DCRUDE_OIL	0.33334	0.5655	0.13271	0.8759	0.28708	0.8346
DGDAXI_R does not Granger Cause DWTI	1.62603	0.2064	4.16416	0.0196	2.67105	0.0546
DWTI does not Granger Cause DGDAXI_R	0.86963	0.3542	0.33244	0.7183	0.33821	0.7978
DDJIA_R does not Granger Cause DWTI	0.06344	0.8019	0.29368	0.7464	0.5449	0.6533
DWTI does not Granger Cause DDJIA_R	0.02556	0.8734	0.9586	0.3885	1.15238	0.3346
DFTSE_R does not Granger Cause DWTI	0.03581	0.8505	0.03133	0.9692	0.07471	0.9734
DWTI does not Granger Cause DFTSE_R	0.0056	0.9406	0.14885	0.862	0.86654	0.463
DFTMIB_R does not Granger Cause DWTI	4.89114	0.0302	2.53924	0.0863	2.78827	0.0474
DWTI does not Granger Cause DFTMIB_R	0.0000049	0.9982	1.33258	0.2705	0.66754	0.575
DIBEX35_R does not Granger Cause DWTI	0.12272	0.7271	0.07298	0.9297	0.26124	0.8531
DWTI does not Granger Cause DIBEX35_R	0.15983	0.6905	1.52354	0.2252	2.46564	0.0699
DJIA_R does not Granger Cause DWTI	0.77312	0.3822	0.76788	0.4679	0.48919	0.691
DWTI does not Granger Cause DJIA_R	0.01227	0.9121	2.70482	0.074	1.46638	0.2317
DNATURAL_GAS does not Granger Cause DWTI	2.57538	0.1129	1.45133	0.2413	1.61733	0.1937
DWTI does not Granger Cause DNATURAL_GAS	6.36455	0.0139	3.0785	0.0524	1.85097	0.1465
DNC_IT does not Granger Cause DWTI	0.00387	0.9506	0.005	0.995	0.01251	0.9981
DWTI does not Granger Cause DNC_IT	0.5675	0.4537	1.34092	0.2683	1.97682	0.126
DND_CH does not Granger Cause DWTI	0.000032	0.9955	0.00029	0.9997	0.00151	0.9999
DWTI does not Granger Cause DND_CH	0.04681	0.8293	0.1231	0.8844	0.10376	0.9576
DND_IT does not Granger Cause DWTI	1.80644	0.1832	1.28712	0.2826	1.13963	0.3396
DWTI does not Granger Cause DND_IT	0.00362	0.9522	1.86469	0.1627	0.91119	0.4405
DLSCO does not Granger Cause DWTI	35.9252	7E-08	22.9478	2E-08	15.107	0.0000001
DWTI does not Granger Cause DLSCO	0.000015	0.997	0.0176	0.9826	0.17202	0.9149
DSPX_R does not Granger Cause DWTI	0.10215	0.7502	0.22766	0.797	0.64805	0.587
DWTI does not Granger Cause DSPX_R	0.06513	0.7993	1.16248	0.3188	1.05948	0.3724
SSE100_R does not Granger Cause DWTI	0.64236	0.4255	0.45326	0.6374	0.25169	0.8599
DWTI does not Granger Cause SSE100_R	0.27143	0.604	1.16251	0.3187	0.83242	0.4808
EUR_CNY does not Granger Cause DWTI	1.77732	0.1867	1.13368	0.3278	0.71534	0.5463
DWTI does not Granger Cause EUR_CNY	0.00131	0.9712	0.27024	0.764	0.30521	0.8215
NC_CH does not Granger Cause DWTI	0.77665	0.3811	0.56075	0.5734	0.4423	0.7235
DWTI does not Granger Cause NC_CH	0.03989	0.8423	0.00733	0.9927	0.02029	0.996
XAU_R does not Granger Cause DWTI	1.00795	0.3188	0.70712	0.4966	0.48752	0.6921
DWTI does not Granger Cause XAU_R	0.03674	0.8485	0.0612	0.9407	1.97534	0.1262
DGDAXI_R does not Granger Cause DCRUDE_OIL	3.48991	0.0658	4.55135	0.0139	3.11406	0.032
DCRUDE_OIL does not Granger Cause DGDAXI_R	3.09507	0.0828	2.02053	0.1404	3.86988	0.013
DDJIA_R does not Granger Cause DCRUDE_OIL	0.48544	0.4882	0.3524	0.7043	1.31087	0.2783
DCRUDE_OIL does not Granger Cause DDJIA_R	1.14294	0.2886	4.4382	0.0154	5.13486	0.003
DFTSE_R does not Granger Cause DCRUDE_OIL	0.44793	0.5055	0.29789	0.7433	0.33352	0.8011
DCRUDE_OIL does not Granger Cause DFTSE_R	0.53018	0.4689	2.14829	0.1244	4.6711	0.0051
DFTMIB_R does not Granger Cause DCRUDE_OIL	7.43633	0.008	3.57113	0.0334	3.52323	0.0196
DCRUDE_OIL does not Granger Cause DFTMIB_R	0.45608	0.5016	5.68559	0.0052	3.28226	0.0262
DIBEX35_R does not Granger Cause DCRUDE_OIL	0.00388	0.9505	0.4571	0.635	0.70906	0.55

DCRUDE_OIL does not Granger Cause DIBEX35_R	0.68443	0.4108	1.81835	0.17	3.32276	0.025
DJIA_R does not Granger Cause DCRUDE_OIL	1.93259	0.1688	1.28783	0.2824	1.31435	0.2772
DCRUDE_OIL does not Granger Cause DJIA_R	0.20664	0.6508	11.9907	0.00003	6.91535	0.0004
DNATURAL_GAS does not Granger Cause DCRUDE_OIL	0.75092	0.3891	0.62846	0.5364	0.7396	0.5322
DCRUDE_OIL does not Granger Cause DNATURAL_GAS	5.32148	0.0239	3.26022	0.0444	2.13306	0.1044
DNC_IT does not Granger Cause DCRUDE_OIL	0.43673	0.5108	0.25329	0.777	0.14997	0.9294
DCRUDE_OIL does not Granger Cause DNC_IT	0.85842	0.3573	6.99476	0.0017	4.52423	0.006
DND_CH does not Granger Cause DCRUDE_OIL	0.000027	0.9959	0.00036	0.9996	0.00102	1
DCRUDE_OIL does not Granger Cause DND_CH	0.04675	0.8294	0.19138	0.8263	0.1409	0.9351
DND_IT does not Granger Cause DCRUDE_OIL	2.16428	0.1456	1.54238	0.2212	1.26425	0.2939
DCRUDE_OIL does not Granger Cause DND_IT	0.81574	0.3694	3.36692	0.0403	2.24293	0.0914
DLSCO does not Granger Cause DCRUDE_OIL	55.5535	2E-10	32.4142	1E-10	21.0619	1E-09
DCRUDE_OIL does not Granger Cause DLSCO	0.08387	0.7729	0.17351	0.8411	0.76365	0.5185
DSPX_R does not Granger Cause DCRUDE_OIL	0.60038	0.441	0.35354	0.7035	1.54724	0.2106
DCRUDE_OIL does not Granger Cause DSPX_R	1.2653	0.2644	4.40458	0.0158	4.64199	0.0053
SSE100_R does not Granger Cause DCRUDE_OIL	0.79141	0.3766	0.50558	0.6054	0.37255	0.7731
DCRUDE_OIL does not Granger Cause SSE100_R	0.00058	0.9809	1.34096	0.2683	1.1591	0.3321
EUR_CNY does not Granger Cause DCRUDE_OIL	1.95867	0.1659	0.99005	0.3768	0.57649	0.6325
DCRUDE_OIL does not Granger Cause EUR_CNY	0.58347	0.4475	0.71801	0.4913	1.5959	0.1987
NC_CH does not Granger Cause DCRUDE_OIL	0.81943	0.3684	0.59704	0.5533	0.50298	0.6815
DCRUDE_OIL does not Granger Cause NC_CH	0.04505	0.8325	0.01568	0.9844	0.05372	0.9835
XAU_R does not Granger Cause DCRUDE_OIL	1.71549	0.1944	0.94641	0.3931	0.71995	0.5436
DCRUDE_OIL does not Granger Cause XAU_R	0.00966	0.922	0.68399	0.508	1.67549	0.1808
DDJIA_R does not Granger Cause DGDAXI_R	2.02381	0.1592	0.30182	0.7404	0.91227	0.4399
DGDAXI_R does not Granger Cause DDJIA_R	19.2245	0.00004	3.47386	0.0365	2.34687	0.0807
DFTSE_R does not Granger Cause DGDAXI_R	0.15996	0.6904	0.33827	0.7142	0.14669	0.9315
DGDAXI_R does not Granger Cause DFTSE_R	2.19778	0.1426	2.83138	0.0658	1.56205	0.2069
DFTMIB_R does not Granger Cause DGDAXI_R	24.5232	0.000005	NA	NA	NA	NA
DGDAXI_R does not Granger Cause DFTMIB_R	2.28164	0.1353	NA	NA	NA	NA
DIBEX35_R does not Granger Cause DGDAXI_R	5.85346	0.0181	5.48144	0.0062	2.3653	0.0789
DGDAXI_R does not Granger Cause DIBEX35_R	1.40797	0.2393	0.61265	0.5448	1.14437	0.3378
DJIA_R does not Granger Cause DGDAXI_R	2.14646	0.1473	4.25992	0.018	3.55824	0.0188
DGDAXI_R does not Granger Cause DJIA_R	16.7319	0.0001	6.05048	0.0038	2.99488	0.037
DNATURAL_GAS does not Granger Cause DGDAXI_R	2.69808	0.1048	4.99053	0.0095	3.07764	0.0335
DGDAXI_R does not Granger Cause DNATURAL_GAS	3.24359	0.0759	1.80741	0.1718	2.16369	0.1006
DNC_IT does not Granger Cause DGDAXI_R	3.43797	0.0678	1.64652	0.2002	3.97768	0.0115
DGDAXI_R does not Granger Cause DNC_IT	0.59501	0.443	0.0814	0.9219	0.04798	0.986
DND_CH does not Granger Cause DGDAXI_R	0.01006	0.9204	0.02639	0.974	0.03935	0.9895
DGDAXI_R does not Granger Cause DND_CH	0.00087	0.9766	0.11559	0.891	0.152	0.928
DND_IT does not Granger Cause DGDAXI_R	1.27997	0.2617	2.8797	0.0629	1.54936	0.21
DGDAXI_R does not Granger Cause DND_IT	3.61271	0.0613	1.61968	0.2054	4.75844	0.0046
DLSCO does not Granger Cause DGDAXI_R	0.16484	0.6859	3.00493	0.0561	3.75742	0.0149

DGDAXI_R does not Granger Cause DLSCO	0.80225	0.3734	0.53944	0.5855	1.6383	0.189
DSPX_R does not Granger Cause DGDAXI_R	1.71057	0.1951	0.52275	0.5952	0.73415	0.5353
DGDAXI_R does not Granger Cause DSPX_R	21.1133	0.00002	3.64118	0.0314	2.46744	0.0698
SSE100_R does not Granger Cause DGDAXI_R	5.76737	0.0189	4.1652	0.0196	3.27016	0.0266
DGDAXI_R does not Granger Cause SSE100_R	0.01296	0.9097	0.01452	0.9856	1.71922	0.1716
EUR_CNY does not Granger Cause DGDAXI_R	0.50923	0.4778	0.36964	0.6923	4.30121	0.0078
DGDAXI_R does not Granger Cause EUR_CNY	8.85636	0.004	3.54938	0.0341	5.51213	0.0019
NC_CH does not Granger Cause DGDAXI_R	0.03692	0.8482	0.02577	0.9746	0.05407	0.9833
DGDAXI_R does not Granger Cause NC_CH	0.00041	0.9839	0.10899	0.8969	0.13106	0.9413
XAU_R does not Granger Cause DGDAXI_R	0.00014	0.9907	6.50284	0.0026	5.26593	0.0026
DGDAXI_R does not Granger Cause XAU_R	1.93297	0.1687	2.96586	0.0581	3.4081	0.0225
DFTSE_R does not Granger Cause DDJIA_R	14.2748	0.0003	1.47223	0.2365	0.93945	0.4267
DDJIA_R does not Granger Cause DFTSE_R	1.93832	0.1681	0.23785	0.789	1.03602	0.3825
DFTMIB_R does not Granger Cause DDJIA_R	19.7361	0.00003	5.05384	0.0089	3.75133	0.015
DDJIA_R does not Granger Cause DFTMIB_R	0.20288	0.6538	0.35659	0.7013	0.62691	0.6002
DIBEX35_R does not Granger Cause DDJIA_R	24.227	0.000005	5.38033	0.0067	3.65992	0.0167
DDJIA_R does not Granger Cause DIBEX35_R	11.7929	0.001	2.70431	0.074	3.51658	0.0198
DJIA_R does not Granger Cause DDJIA_R	30.4871	0.0000005	NA	NA	NA	NA
DDJIA_R does not Granger Cause DJIA_R	4.19757	0.0441	NA	NA	NA	NA
DNATURAL_GAS does not Granger Cause DDJIA_R	0.57406	0.4511	3.67162	0.0305	2.33601	0.0817
DDJIA_R does not Granger Cause DNATURAL_GAS	1.30893	0.2564	1.10943	0.3356	1.13681	0.3407
DNC_IT does not Granger Cause DDJIA_R	8.343	0.0051	1.64915	0.1997	2.27506	0.088
DDJIA_R does not Granger Cause DNC_IT	4.88918	0.0302	2.24186	0.1139	2.40846	0.0749
DND_CH does not Granger Cause DDJIA_R	0.01229	0.912	0.01122	0.9888	0.00756	0.9991
DDJIA_R does not Granger Cause DND_CH	0.01568	0.9007	0.01215	0.9879	0.0763	0.9726
DND_IT does not Granger Cause DDJIA_R	1.84108	0.1791	0.65972	0.5202	0.54317	0.6545
DDJIA_R does not Granger Cause DND_IT	0.12629	0.7233	5.14002	0.0083	7.20817	0.0003
DLSCO does not Granger Cause DDJIA_R	0.26288	0.6097	0.78767	0.4589	2.82084	0.0456
DDJIA_R does not Granger Cause DLSCO	2.91316	0.0922	3.50326	0.0356	4.0529	0.0105
DSPX_R does not Granger Cause DDJIA_R	0.48539	0.4882	0.58971	0.5573	0.93295	0.4298
DDJIA_R does not Granger Cause DSPX_R	0.05804	0.8103	0.08375	0.9198	0.79063	0.5034
SSE100_R does not Granger Cause DDJIA_R	10.408	0.0019	3.97076	0.0233	2.59763	0.0596
DDJIA_R does not Granger Cause SSE100_R	1.05331	0.3082	0.64063	0.5301	1.8856	0.1406
EUR_CNY does not Granger Cause DDJIA_R	0.14185	0.7076	0.82076	0.4443	4.25907	0.0082
DDJIA_R does not Granger Cause EUR_CNY	25.7438	0.000003	11.6983	0.00004	9.94987	0.00002
NC_CH does not Granger Cause DDJIA_R	0.01885	0.8912	0.01587	0.9843	0.02449	0.9948
DDJIA_R does not Granger Cause NC_CH	0.01506	0.9027	0.01101	0.989	0.06645	0.9775
XAU_R does not Granger Cause DDJIA_R	0.286	0.5944	1.13947	0.3259	0.87906	0.4566
DDJIA_R does not Granger Cause XAU_R	4.26441	0.0425	6.96113	0.0018	5.35932	0.0023
DFTMIB_R does not Granger Cause DFTSE_R	15.5429	0.0002	4.67352	0.0125	3.65167	0.0169
DFTSE_R does not Granger Cause DFTMIB_R	2.13102	0.1487	0.63372	0.5337	0.35416	0.7863
DIBEX35_R does not Granger Cause DFTSE_R	11.7367	0.001	6.74773	0.0021	3.99477	0.0112

DFTSE_R does not Granger Cause DIBEX35_R	19.8855	0.00003	10.2522	0.0001	6.42742	0.0007
DJIA_R does not Granger Cause DFTSE_R	4.41575	0.0391	5.17402	0.0081	3.404	0.0226
DFTSE_R does not Granger Cause DJIA_R	11.2824	0.0013	3.56226	0.0337	1.62933	0.191
DNATURAL_GAS does not Granger Cause DFTSE_R	4.93847	0.0294	7.55888	0.0011	4.43906	0.0067
DFTSE_R does not Granger Cause DNATURAL_GAS	2.32656	0.1316	1.39208	0.2555	2.70531	0.0524
DNC_IT does not Granger Cause DFTSE_R	2.95784	0.0898	0.74778	0.4772	1.86651	0.1438
DFTSE_R does not Granger Cause DNC_IT	2.59274	0.1117	2.48971	0.0904	2.44043	0.0721
DND_CH does not Granger Cause DFTSE_R	0.06948	0.7928	0.05505	0.9465	0.04725	0.9863
DFTSE_R does not Granger Cause DND_CH	0.00054	0.9815	0.08663	0.9171	0.09568	0.9622
DND_IT does not Granger Cause DFTSE_R	1.1242	0.2926	1.62113	0.2051	1.13041	0.3432
DFTSE_R does not Granger Cause DND_IT	3.40425	0.0691	1.45758	0.2399	4.29499	0.0079
DLSCO does not Granger Cause DFTSE_R	0.0576	0.811	2.20191	0.1183	3.97688	0.0115
DFTSE_R does not Granger Cause DLSCO	2.63216	0.1091	1.64007	0.2014	1.30853	0.2791
DSPX_R does not Granger Cause DFTSE_R	1.38584	0.243	0.47285	0.6252	0.8447	0.4743
DFTSE_R does not Granger Cause DSPX_R	14.7947	0.0003	1.29385	0.2808	0.79526	0.5009
SSE100_R does not Granger Cause DFTSE_R	9.54749	0.0028	5.002	0.0094	3.19387	0.0291
DFTSE_R does not Granger Cause SSE100_R	0.27028	0.6047	0.19223	0.8256	1.446	0.2374
EUR_CNY does not Granger Cause DFTSE_R	0.62828	0.4306	0.19161	0.8261	3.52012	0.0197
DFTSE_R does not Granger Cause EUR_CNY	16.6735	0.0001	7.01834	0.0017	6.79576	0.0005
NC_CH does not Granger Cause DFTSE_R	0.01056	0.9184	0.04854	0.9526	0.03194	0.9923
DFTSE_R does not Granger Cause NC_CH	0.00037	0.9846	0.09834	0.9065	0.09516	0.9625
XAU_R does not Granger Cause DFTSE_R	0.0223	0.8817	6.61847	0.0023	4.87305	0.004
DFTSE_R does not Granger Cause XAU_R	1.56672	0.2147	4.49184	0.0147	5.28781	0.0025
DIBEX35_R does not Granger Cause DFTMIB_R	8.82457	0.004	3.67991	0.0303	2.96382	0.0384
DFTMIB_R does not Granger Cause DIBEX35_R	4.53097	0.0367	3.44393	0.0375	2.50402	0.0667
DJIA_R does not Granger Cause DFTMIB_R	0.0016	0.9682	0.28714	0.7513	0.44827	0.7193
DFTMIB_R does not Granger Cause DJIA_R	0.01825	0.8929	8.08338	0.0007	4.85237	0.0041
DNATURAL_GAS does not Granger Cause DFTMIB_R	3.73389	0.0573	5.03988	0.0091	3.05921	0.0342
DFTMIB_R does not Granger Cause DNATURAL_GAS	2.65813	0.1074	1.75077	0.1813	1.95729	0.129
DNC_IT does not Granger Cause DFTMIB_R	0.05257	0.8193	0.16908	0.8448	1.83906	0.1486
DFTMIB_R does not Granger Cause DNC_IT	1.76672	0.188	1.18036	0.3133	1.04829	0.3772
DND_CH does not Granger Cause DFTMIB_R	0.000042	0.9948	0.06194	0.94	0.03784	0.9901
DFTMIB_R does not Granger Cause DND_CH	0.04294	0.8364	0.04892	0.9523	0.09172	0.9644
DND_IT does not Granger Cause DFTMIB_R	1.80723	0.1831	1.54995	0.2196	1.61776	0.1936
DFTMIB_R does not Granger Cause DND_IT	0.10253	0.7497	2.39514	0.0987	2.90057	0.0414
DLSCO does not Granger Cause DFTMIB_R	2.06788	0.1548	2.41525	0.0969	4.63093	0.0053
DFTMIB_R does not Granger Cause DLSCO	0.31838	0.5743	0.39991	0.6719	0.32891	0.8045
DSPX_R does not Granger Cause DFTMIB_R	0.3269	0.5693	0.25185	0.7781	0.67842	0.5684
DFTMIB_R does not Granger Cause DSPX_R	21.2133	0.00002	5.36619	0.0068	3.89639	0.0126
SSE100_R does not Granger Cause DFTMIB_R	0.3005	0.5852	0.46279	0.6314	0.56648	0.639
DFTMIB_R does not Granger Cause SSE100_R	4.13707	0.0456	4.10012	0.0207	4.18962	0.0089
EUR_CNY does not Granger Cause DFTMIB_R	0.06545	0.7988	0.11604	0.8906	2.2824	0.087

DFTMIB_R does not Granger Cause EUR_CNY	15.3431	0.0002	7.8432	0.0008	7.01151	0.0004
NC_CH does not Granger Cause DFTMIB_R	0.19227	0.6623	0.06595	0.9362	0.04663	0.9865
DFTMIB_R does not Granger Cause NC_CH	0.25002	0.6186	0.21448	0.8075	0.13551	0.9385
XAU_R does not Granger Cause DFTMIB_R	5.69148	0.0196	4.67835	0.0124	4.59801	0.0055
DFTMIB_R does not Granger Cause XAU_R	0.00709	0.9331	3.73531	0.0287	2.80705	0.0462
DJIA_R does not Granger Cause DIBEX35_R	0.08931	0.7659	8.4625	0.0005	6.7593	0.0005
DIBEX35_R does not Granger Cause DJIA_R	22.8461	0.000009	8.57193	0.0005	4.83086	0.0042
DNATURAL_GAS does not Granger Cause DIBEX35_R	6.09195	0.016	8.98252	0.0003	5.7801	0.0014
DIBEX35_R does not Granger Cause DNATURAL_GAS	1.59014	0.2114	0.80711	0.4503	2.30686	0.0847
DNC_IT does not Granger Cause DIBEX35_R	3.98298	0.0497	1.82626	0.1687	2.29086	0.0863
DIBEX35_R does not Granger Cause DNC_IT	2.38967	0.1265	2.72452	0.0726	2.24478	0.0912
DND_CH does not Granger Cause DIBEX35_R	0.00011	0.9915	0.05718	0.9445	0.04495	0.9872
DIBEX35_R does not Granger Cause DND_CH	0.00235	0.9615	0.03658	0.9641	0.06157	0.9798
DND_IT does not Granger Cause DIBEX35_R	0.34712	0.5576	1.24242	0.2951	1.02918	0.3855
DIBEX35_R does not Granger Cause DND_IT	2.4936	0.1187	1.78862	0.1749	3.89499	0.0126
DLSCO does not Granger Cause DIBEX35_R	3.90236	0.0521	3.97109	0.0233	5.15146	0.0029
DIBEX35_R does not Granger Cause DLSCO	5.47177	0.0221	4.41645	0.0157	3.11121	0.0321
DSPX_R does not Granger Cause DIBEX35_R	11.0469	0.0014	2.38596	0.0996	3.29831	0.0257
DIBEX35_R does not Granger Cause DSPX_R	25.2302	0.000004	5.18958	0.008	3.49572	0.0203
SSE100_R does not Granger Cause DIBEX35_R	6.66637	0.0119	8.27035	0.0006	5.37335	0.0023
DIBEX35_R does not Granger Cause SSE100_R	0.00111	0.9735	0.06023	0.9416	1.44208	0.2385
EUR_CNY does not Granger Cause DIBEX35_R	6.01201	0.0166	2.8411	0.0652	5.29375	0.0025
DIBEX35_R does not Granger Cause EUR_CNY	17.1513	0.00009	8.16609	0.0007	5.86456	0.0013
NC_CH does not Granger Cause DIBEX35_R	0.00055	0.9814	0.00037	0.9996	0.04437	0.9875
DIBEX35_R does not Granger Cause NC_CH	0.00018	0.9893	0.03873	0.962	0.04719	0.9863
XAU_R does not Granger Cause DIBEX35_R	2.44185	0.1225	13.2489	0.00001	9.1124	0.00004
DIBEX35_R does not Granger Cause XAU_R	2.9133	0.0922	5.23048	0.0077	6.38535	0.0007
DNATURAL_GAS does not Granger Cause DJIA_R	4.0687	0.0474	3.03054	0.0548	2.39317	0.0763
DJIA_R does not Granger Cause DNATURAL_GAS	1.57428	0.2136	1.00863	0.37	0.69601	0.5578
DNC_IT does not Granger Cause DJIA_R	0.06244	0.8034	1.14317	0.3248	0.91637	0.4379
DJIA_R does not Granger Cause DNC_IT	5.36566	0.0234	2.35201	0.1028	2.3769	0.0778
DND_CH does not Granger Cause DJIA_R	0.000098	0.9921	0.03232	0.9682	0.02163	0.9956
DJIA_R does not Granger Cause DND_CH	0.03852	0.845	0.02501	0.9753	0.02723	0.9939
DND_IT does not Granger Cause DJIA_R	0.59426	0.4433	0.40947	0.6656	0.32348	0.8084
DJIA_R does not Granger Cause DND_IT	0.00332	0.9542	0.00795	0.9921	3.88076	0.0128
DLSCO does not Granger Cause DJIA_R	0.34167	0.5607	0.31661	0.7297	6.06408	0.001
DJIA_R does not Granger Cause DLSCO	1.3975	0.241	1.65485	0.1986	2.83408	0.0448
DSPX_R does not Granger Cause DJIA_R	4.5874	0.0356	1.73876	0.1833	0.62414	0.6019
DJIA_R does not Granger Cause DSPX_R	27.9568	0.000001	5.85658	0.0045	4.20102	0.0088
SSE100_R does not Granger Cause DJIA_R	0.10887	0.7424	0.69733	0.5013	0.47661	0.6996
DJIA_R does not Granger Cause SSE100_R	4.34363	0.0406	3.12198	0.0503	2.5813	0.0607
EUR_CNY does not Granger Cause DJIA_R	5.48669	0.0219	1.32968	0.2712	4.65458	0.0051

DJIA_R does not Granger Cause EUR_CNY	34.5284	0.0000001	14.6014	0.000005	10.9528	0.000006
NC_CH does not Granger Cause DJIA_R	0.12137	0.7286	0.041	0.9599	0.01943	0.9963
DJIA_R does not Granger Cause NC_CH	0.12077	0.7292	0.09268	0.9116	0.06255	0.9794
XAU_R does not Granger Cause DJIA_R	7.7373	0.0069	3.49038	0.0359	3.69683	0.0159
DJIA_R does not Granger Cause XAU_R	1.27667	0.2622	4.03297	0.022	4.40173	0.0069
DNC_IT does not Granger Cause DNATURAL_GAS	0.14623	0.7033	0.0076	0.9924	0.32145	0.8098
DNATURAL_GAS does not Granger Cause DNC_IT	5.70832	0.0195	2.9576	0.0586	1.99555	0.1232
DND_CH does not Granger Cause DNATURAL_GAS	0.00405	0.9494	1.14548	0.324	1.47552	0.2293
DNATURAL_GAS does not Granger Cause DND_CH	0.71795	0.3996	0.32552	0.7233	1.2013	0.3162
DND_IT does not Granger Cause DNATURAL_GAS	3.23609	0.0762	2.27477	0.1105	1.46149	0.2331
DNATURAL_GAS does not Granger Cause DND_IT	1.9116	0.1711	1.02305	0.3649	1.13549	0.3412
DLSCO does not Granger Cause DNATURAL_GAS	9.4399	0.003	8.61552	0.0005	5.86559	0.0013
DNATURAL_GAS does not Granger Cause DLSCO	0.00848	0.9269	0.00603	0.994	0.05207	0.9842
DSPX_R does not Granger Cause DNATURAL_GAS	1.41539	0.2381	1.11757	0.3329	1.25479	0.2971
DNATURAL_GAS does not Granger Cause DSPX_R	0.84894	0.3599	3.34795	0.041	2.13607	0.104
SSE100_R does not Granger Cause DNATURAL_GAS	1.25393	0.2665	1.27236	0.2867	1.08788	0.3605
DNATURAL_GAS does not Granger Cause SSE100_R	1.19737	0.2775	1.05447	0.3539	0.78664	0.5056
EUR_CNY does not Granger Cause DNATURAL_GAS	3.26436	0.075	1.98866	0.1446	1.33854	0.2694
DNATURAL_GAS does not Granger Cause EUR_CNY	0.05276	0.819	0.35714	0.701	0.32804	0.8051
NC_CH does not Granger Cause DNATURAL_GAS	0.13446	0.7149	0.18404	0.8323	1.24256	0.3014
DNATURAL_GAS does not Granger Cause NC_CH	1.04031	0.3112	0.44604	0.642	1.15101	0.3352
XAU_R does not Granger Cause DNATURAL_GAS	4.13711	0.0456	2.62288	0.0798	1.89999	0.1382
DNATURAL_GAS does not Granger Cause XAU_R	0.58939	0.4452	0.20339	0.8164	0.40088	0.7528
DND_CH does not Granger Cause DNC_IT	0.000029	0.9957	0.0000094	1	0.00134	0.9999
DNC_IT does not Granger Cause DND_CH	0.000078	0.993	0.000047	1	0.000067	1
DND_IT does not Granger Cause DNC_IT	2.15811	0.1462	2.6905	0.075	1.91646	0.1355
DNC_IT does not Granger Cause DND_IT	0.00286	0.9575	4.02266	0.0223	5.57975	0.0018
DLSCO does not Granger Cause DNC_IT	2.76031	0.101	1.72452	0.1859	5.8209	0.0014
DNC_IT does not Granger Cause DLSCO	0.31036	0.5792	0.18924	0.828	0.3604	0.7818
DSPX_R does not Granger Cause DNC_IT	5.2001	0.0255	2.24586	0.1135	2.32974	0.0824
DNC_IT does not Granger Cause DSPX_R	10.765	0.0016	2.23276	0.1149	2.42336	0.0736
SSE100_R does not Granger Cause DNC_IT	1.4542	0.2318	1.5875	0.2118	1.87237	0.1428
DNC_IT does not Granger Cause SSE100_R	3.18273	0.0786	2.67617	0.076	2.06989	0.1126
EUR_CNY does not Granger Cause DNC_IT	0.75398	0.3881	2.34397	0.1035	1.51244	0.2194
DNC_IT does not Granger Cause EUR_CNY	0.57477	0.4508	0.81844	0.4454	0.81673	0.4892
NC_CH does not Granger Cause DNC_IT	0.11465	0.7359	0.09985	0.9051	0.07325	0.9741
DNC_IT does not Granger Cause NC_CH	0.02347	0.8787	0.01261	0.9875	0.0118	0.9982
XAU_R does not Granger Cause DNC_IT	1.77431	0.1871	1.34858	0.2664	1.15902	0.3321
DNC_IT does not Granger Cause XAU_R	1.44568	0.2332	1.768	0.1783	1.6365	0.1894
DND_IT does not Granger Cause DND_CH	0.000022	0.9962	0.000008	1	0.000028	1
DND_CH does not Granger Cause DND_IT	0.00013	0.991	0.00016	0.9998	0.00015	1
DLSCO does not Granger Cause DND_CH	0.01634	0.8986	0.04048	0.9604	0.24063	0.8677

DND_CH does not Granger Cause DLSCO	0.02857	0.8662	0.02025	0.98	0.04	0.9892
DSPX_R does not Granger Cause DND_CH	0.00297	0.9567	0.00222	0.9978	0.0421	0.9884
DND_CH does not Granger Cause DSPX_R	0.00026	0.9871	0.01475	0.9854	0.01069	0.9985
SSE100_R does not Granger Cause DND_CH	0.04824	0.8268	0.26009	0.7717	0.36761	0.7766
DND_CH does not Granger Cause SSE100_R	1.13669	0.2899	0.65103	0.5247	0.65127	0.585
EUR_CNY does not Granger Cause DND_CH	0.09353	0.7606	0.06083	0.941	0.06099	0.9801
DND_CH does not Granger Cause EUR_CNY	0.00504	0.9436	0.02484	0.9755	0.10162	0.9588
NC_CH does not Granger Cause DND_CH	11.2291	0.0013	NA	NA	NA	NA
DND_CH does not Granger Cause NC_CH	4.4127	0.0392	NA	NA	NA	NA
XAU_R does not Granger Cause DND_CH	0.0327	0.857	0.03085	0.9696	0.08525	0.9679
DND_CH does not Granger Cause XAU_R	0.11138	0.7395	0.31473	0.731	0.23114	0.8744
DLSCO does not Granger Cause DND_IT	6.57734	0.0124	3.75535	0.0283	3.39436	0.0229
DND_IT does not Granger Cause DLSCO	0.67079	0.4155	0.68645	0.5068	0.46457	0.708
DSPX_R does not Granger Cause DND_IT	0.09183	0.7627	5.36107	0.0069	6.81524	0.0005
DND_IT does not Granger Cause DSPX_R	1.35856	0.2476	0.38943	0.6789	0.3688	0.7758
SSE100_R does not Granger Cause DND_IT	0.12743	0.7222	1.71176	0.1881	1.27148	0.2914
DND_IT does not Granger Cause SSE100_R	0.24031	0.6255	0.12002	0.8871	0.87427	0.459
EUR_CNY does not Granger Cause DND_IT	0.29555	0.5884	0.37403	0.6893	0.46632	0.7068
DND_IT does not Granger Cause EUR_CNY	3.83749	0.054	2.60063	0.0815	1.67336	0.1812
NC_CH does not Granger Cause DND_IT	0.24026	0.6255	0.11818	0.8887	0.04698	0.9864
DND_IT does not Granger Cause NC_CH	0.0457	0.8313	0.04246	0.9585	0.0344	0.9914
XAU_R does not Granger Cause DND_IT	3.16454	0.0795	2.20745	0.1177	3.99518	0.0112
DND_IT does not Granger Cause XAU_R	2.45655	0.1214	1.73256	0.1844	1.3591	0.263
DSPX_R does not Granger Cause DLSCO	2.95454	0.0899	3.72306	0.0291	4.24025	0.0084
DLSCO does not Granger Cause DSPX_R	0.33126	0.5667	1.00162	0.3726	2.86899	0.043
SSE100_R does not Granger Cause DLSCO	0.00185	0.9658	0.00169	0.9983	0.50287	0.6816
DLSCO does not Granger Cause SSE100_R	1.25257	0.2668	0.57762	0.5639	1.21256	0.3121
EUR_CNY does not Granger Cause DLSCO	0.03557	0.8509	1.61451	0.2064	1.27628	0.2898
DLSCO does not Granger Cause EUR_CNY	3.39143	0.0697	4.54547	0.014	3.23325	0.0278
NC_CH does not Granger Cause DLSCO	1.68844	0.198	1.02971	0.3625	0.66915	0.574
DLSCO does not Granger Cause NC_CH	0.67526	0.4139	0.28267	0.7546	0.18786	0.9043
XAU_R does not Granger Cause DLSCO	2.40403	0.1254	1.53583	0.2225	1.19692	0.3178
DLSCO does not Granger Cause XAU_R	0.12838	0.7212	0.83923	0.4364	1.6284	0.1912
SSE100_R does not Granger Cause DSPX_R	10.5509	0.0018	4.11792	0.0204	2.64269	0.0565
DSPX_R does not Granger Cause SSE100_R	0.87229	0.3534	0.50506	0.6057	1.91497	0.1357
EUR_CNY does not Granger Cause DSPX_R	0.2816	0.5973	0.68421	0.5079	3.95873	0.0117
DSPX_R does not Granger Cause EUR_CNY	25.692	0.000003	11.6227	0.00004	9.44451	0.00003
NC_CH does not Granger Cause DSPX_R	0.00605	0.9382	0.01081	0.9892	0.02536	0.9945
DSPX_R does not Granger Cause NC_CH	0.00236	0.9614	0.00083	0.9992	0.0337	0.9916
XAU_R does not Granger Cause DSPX_R	0.40148	0.5283	1.12126	0.3317	0.90945	0.4413
DSPX_R does not Granger Cause XAU_R	4.6375	0.0346	6.89006	0.0019	5.99323	0.0011
EUR_CNY does not Granger Cause SSE100_R	0.66546	0.4173	0.35047	0.7056	0.28518	0.8359

SSE100_R does not Granger Cause EUR_CNY	3.70951	0.058	2.76639	0.0698	2.70096	0.0525
NC_CH does not Granger Cause SSE100_R	0.52434	0.4713	1.67175	0.1953	1.13251	0.3423
SSE100_R does not Granger Cause NC_CH	0.30891	0.58	0.1441	0.8661	0.09123	0.9646
XAU_R does not Granger Cause SSE100_R	6.9399	0.0103	3.47216	0.0365	4.62719	0.0053
SSE100_R does not Granger Cause XAU_R	0.4711	0.4947	1.32488	0.2724	1.17442	0.3261
NC_CH does not Granger Cause EUR_CNY	0.01545	0.9014	0.00574	0.9943	0.02867	0.9934
EUR_CNY does not Granger Cause NC_CH	0.15987	0.6904	0.10455	0.9009	0.09572	0.9621
XAU_R does not Granger Cause EUR_CNY	6.40469	0.0135	3.49534	0.0357	2.32203	0.083
EUR_CNY does not Granger Cause XAU_R	1.35288	0.2486	5.58045	0.0056	3.4705	0.0208
XAU_R does not Granger Cause NC_CH	0.0473	0.8284	0.01668	0.9835	0.04828	0.9858
NC_CH does not Granger Cause XAU_R	0.02545	0.8737	0.06236	0.9396	0.2484	0.8622

Source: authors' own calculations. Notes: for the definition of variables, please see Table 1.
